# Flubendazole elicits anti-cancer effects via targeting EVA1A-modulated autophagy and apoptosis in Triple-negative Breast Cancer

**DOI:** 10.7150/thno.43473

**Published:** 2020-07-02

**Authors:** Yongqi Zhen, Rongyan Zhao, Minjuan Wang, Xing Jiang, Feng Gao, Leilei Fu, Lan Zhang, Xian-Li Zhou

**Affiliations:** 1 School of Life Science and Engineering, Southwest Jiaotong University, Chengdu, P.R. China.; 2 Key Laboratory of Advanced Technologies of Materials, Ministry of Education, Southwest Jiaotong University, Chengdu, P.R. China.

**Keywords:** Flubendazole, Triple-negative breast cancer, Autophagic cell death, Cell migration, EVA1A

## Abstract

**Background:** Triple-negative breast cancer (TNBC) is one of the most prevalent neoplastic diseases worldwide, but efficacious treatments for this pathological condition are still challenging. The lack of an effective targeted therapy also leads to a poor prognosis for patients affected by TNBC. In the present study, we repurposed the distinctive inhibitory effects of flubendazole, a traditional anthelmintic drug, towards the putative modulation of proliferation and migration of TNBC *in vitro and in vivo*.

**Methods:** According to a series of experimental approaches, including immunofluorescence (IF), immunoblotting (IB), siRNA and GFP-mRFP-LC3 plasmid transfection, respectively, we have found that flubendazole is capable of inducing autophagic cell death and apoptosis, thus exerting some anti-proliferative and anti-migration activity in TNBC cells. The therapeutic effects of flubendazole were evaluated by xenograft mouse models, followed by immunohistochemistry (IHC), IF and IB. Changes in the gene expression profiles of flubendazole-treated TNBC cells were analyzed by RNA sequencing (RNA-seq) and validated by IB. The potential binding mode of flubendazole and EVA1A was predicted by molecular docking and demonstrated by site-directed mutagenesis.

**Results:** We have presently found that flubendazole exhibits a considerable anti-proliferative activity *in vitro* and *in vivo*. Mechanistically, the induction of autophagic cell death appears to be pivotal for flubendazole-mediated growth inhibition of TNBC cells, whereas blocking autophagy was able to improve the survival rate and migration ability of flubendazole-treated TNBC cells. Specifically, RNA-seq analysis showed that flubendazole treatment could promote the up-regulation of EVA1A. Flubendazole may regulate autophagy and apoptosis by targeting EVA1A, thus affecting the mechanisms of TNBC proliferation and migration. Furthermore, Thr113 may be the key amino acid residues for the binding of flubendazole to EVA1A.

**Conclusion:** Our results provide novel insights towards the putative anti-cancer efficacy of flubendazole. Furthermore, here we show that flubendazole could serve as a potential therapeutic drug in TNBC. Altogether, this study highlights the possibility of this repurposed autophagic inducer for future cancer treatments.

## Introduction

Breast cancer is one of the most common malignancies worldwide, and is also considered the leading cause of cancer-related death among women 1-2. Triple-negative breast cancer (TNBC) is a heterogeneous subtype of breast cancer with poor clinical outcome, which is characterized by the lack of three targeted proteins: estrogen receptor (ER), progesterone receptor (PR) and human epidermal growth factor receptor-2 (HER-2) 3. TNBC is characterized by a high risk of distant recurrence and metastasis in the first 3-5 years after diagnosis, where lung metastasis is one of its main manifestations 4. A number of clinical strategies, including modified chemotherapeutic approaches, targeted DNA damage response, angiogenesis inhibitors, immunological checkpoint inhibitors, and anti-androgens, have been currently investigated in phase 1-3 studies 5-7. Still, due to the concurrent absence of the three major hormone-related receptors, therapeutic targeting of TNBC is still challenging. In fact, TNBC patients are frequently less responsive to conventional hormonal or trastuzumab-based therapies, which ultimately results in a comparatively higher mortality rate 8. Hence, the identification of novel therapeutic agents for the effective treatment of TNBC is warranted.

Autophagy is a self-regulating process in which aggregated proteins and aged (or malfunctioning) organelles are separated by double-membrane autophagosomes and then degraded in autolysosomes. This “recycling” process enables cells to cope with various stress conditions and maintain cellular homeostasis 9-10. Autophagy participates in the progression of a number of disorders, such as cancer, auto-immune diseases, infection and neurodegeneration 11-12. In the context of cancer progression, it is worth noting that autophagy is considered a “double-edged sword”. Anti-cancer treatment can usually induce autophagy to prolong the cancer cell survival, by removing damaged organelles and thus recovering nutrients after anti-cancer treatment 13. However, a series of small molecule compounds targeting autophagy-related proteins (or autophagic processes) have shown distinct effects in cancer cells, eventually leading to (i) cytotoxicity, (ii) inhibition of cell proliferation, (iii) induction of autophagic cell death and/or (iv) apoptosis 14.

Flubendazole, a broad-spectrum anthelmintic drug belonging to the benzimidazole group, has been repurposed as a promising anti-cancer agent. A number of studies have demonstrated that flubendazole may exert an anti-proliferative activity in different types of malignancies, including leukemia, myeloma, melanoma, oral squamous carcinoma, esophageal squamous carcinoma, neuroblastoma, liver, colorectal and breast cancers 15-27. We have previously reported that flubendazole may induce autophagy by activating Atg4B in TNBC cells 28. Screening analyses using HeLa cells have also shown that flubendazole acts as a potent inducer of autophagy flux by affecting acetylated and dynamic microtubules, in a reciprocal manner 29. However, the intricate autophagy-related mechanisms underpinning the anti-cancer effect of flubendazole remain to be further defined. To our knowledge, very limited data has been generated in regard to the induction of autophagy in TNBC by flubendazole.

In the present study, we have characterized the inhibitory effect of flubendazole on the proliferation and migration of TNBC cells *in vitro* and *in vivo*. Specifically, we observed that flubendazole may induce autophagic cell death in TNBC. RNA-seq analyses confirmed that flubendazole can up-regulate the expression of EVA1A, a protein-coding gene involved in autophagy and apoptosis-inducing cell death 30. We further demonstrated that flubendazole has a therapeutic potential by targeting EVA1A-mediated cell death, which is associated with autophagy and apoptosis in TNBC. Together, these findings shed light towards the exploration of unexpected roles of flubendazole in autophagy, thus providing pre-clinical evidence for a novel therapeutic strategy in TNBC.

## Results

### Flubendazole inhibits the proliferation of TNBC *in vivo* and *in vitro*

**Figure 1:**
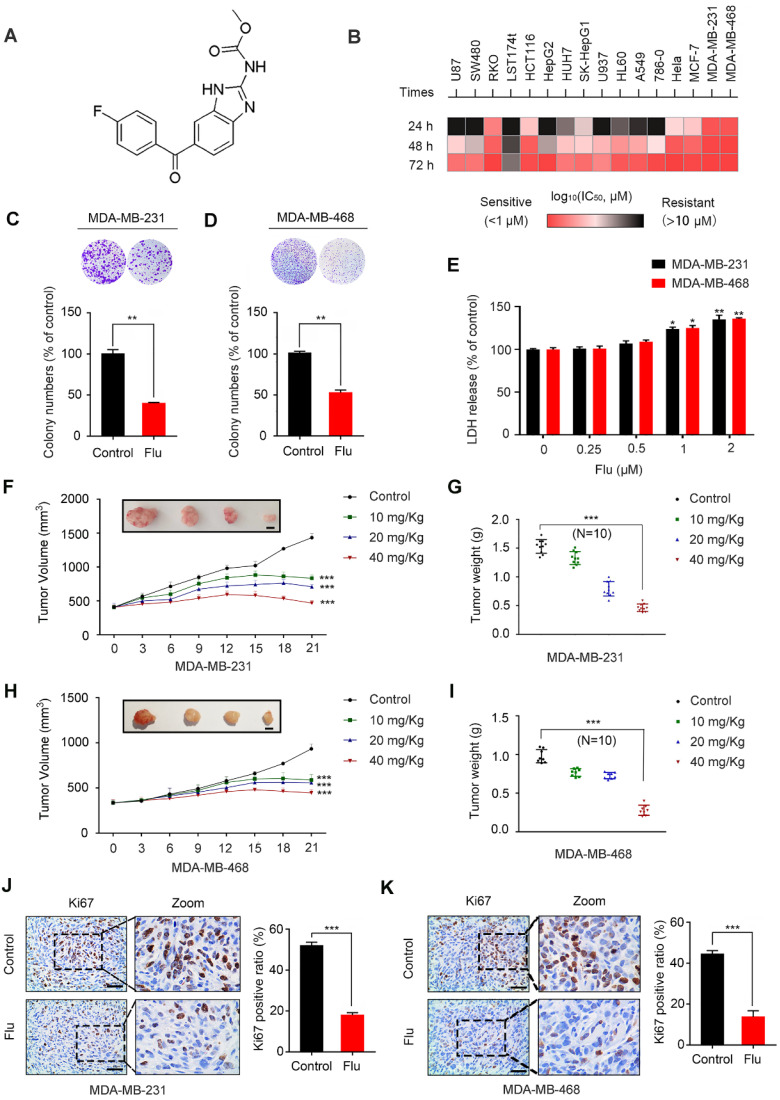
**Flubendazole inhibits TNBC *in vitro* and *in vivo*.** (**A**) The chemical structure of flubendazole. (**B**) Heat map of mean IC_50_ values of flubendazole in multiple tumor cell lines at different times. Cell viabilities were measured by MTT assay, and the IC_50_ values were calculated by Prism 7.0. (**C-D**) Colony formation assay of MDA-MB-231 and MDA-MB-468 cells treated with or without flubendazole (0.5 µM). Representative images and quantification of colonies were shown. (**E**) LDH release assay of MDA-MB-231 and MDA-MB-468 cells treated with the indicated concentrations of flubendazole for 24 h. (**F-I**) The images of isolated tumors derived from mice and antitumor activities of flubendazole in representative MDA-MB-231 and MDA-MB-468 tumors from mice after vehicle and flubendazole treatment: (F and H) tumor volume and (G and I) tumor weight (n = 10), Scale bar, 0.5 cm. (**J-K**) The expression of Ki67 in representative MDA-MB-231 and MDA-MB-468 tumor sections of nude mice from vehicle and median dose (20 mg/Kg). Representative images and quantitative analysis of the percentage of positive ratios were shown. Scale bar, 40 µm. Data are expressed as mean ± SEM. All data were representative of at least three independent experiments. *, *P* < 0.05, **, *P* < 0.01, ***, *P* < 0.001. Statistical significance compared with respective control groups.

The chemical structure of flubendazole is depicted in Figure 1A. To validate the potential anti-cancer activity of flubendazole, MTT assays were conducted to assess the growth of multiple tumor cell lines following flubendazole treatment. The viability of cancer cells treated with flubendazole was significantly reduced in a time- and dose-dependent manner (Figure 1B and Figure S1A-C). Notably, the TNBC cell lines MDA-MB-231 and MDA-MB-468 were more sensitive to flubendazole within 24 h treatment (IC_50_ values of 0.623 μM and 0.728 μM, respectively). Accordingly, a concentration of 0.5 μM flubendazole was used in subsequent *in vitro* experiments. Consistently, the proliferation of TNBC cells was significantly inhibited after flubendazole treatment, as evidenced by a reduced colony formation (Figure 1C and Figure 1D). We then performed LDH (lactate dehydrogenase) release assay and found high concentrations of flubendazole exhibited certain cytotoxicity in MDA-MB-231 and MDA-MB-468 cells (Figure 1E). Subsequently, we examined whether flubendazole had any cytotoxic effect in normal MCF10A human breast cells and found the tested dose of flubendazole (0.5 μM) did not cause any obvious toxicity to MCF10A cells (Figure S2). Taken together, these results suggest that flubendazole acts as an anti-cancer agent *in vitro*, especially against TNBC.

To ascertain the antineoplastic effects of flubendazole *in vivo*, a mouse xenograft tumor model was generated by subcutaneously inoculating MDA-MB-231 and MDA-MB-468 cells into nude mice. According to the measured tumor volume and weight, we found that xenografted tumors treated with flubendazole grew at a slower rate than those treated with placebo (Figure 1F-I). Using Ki67 as a clinical marker to assess cancer proliferation, we verified that flubendazole treatment consistently led to a weaker Ki67 staining as compared with the control group (Figure 1J-K and Figure S3A). Moreover, no significant difference in the behavior, feeding pattern and overall activity of control and flubendazole-treated mice was observed during the whole experiment. Furthermore, flubendazole had no significant effect on the mice body weight at the higher doses (Figure S4A). H&E staining of the major tissues, such as heart, liver, spleen, lung, and kidney, also showed that flubendazole had no apparent toxicity to the mice (Figure S4B). Collectively, these findings demonstrate that flubendazole can effectively inhibit proliferation of TNBC *in vitro* and *in vivo*.

### Flubendazole induces apoptosis in TNBC cells

**Figure 2:**
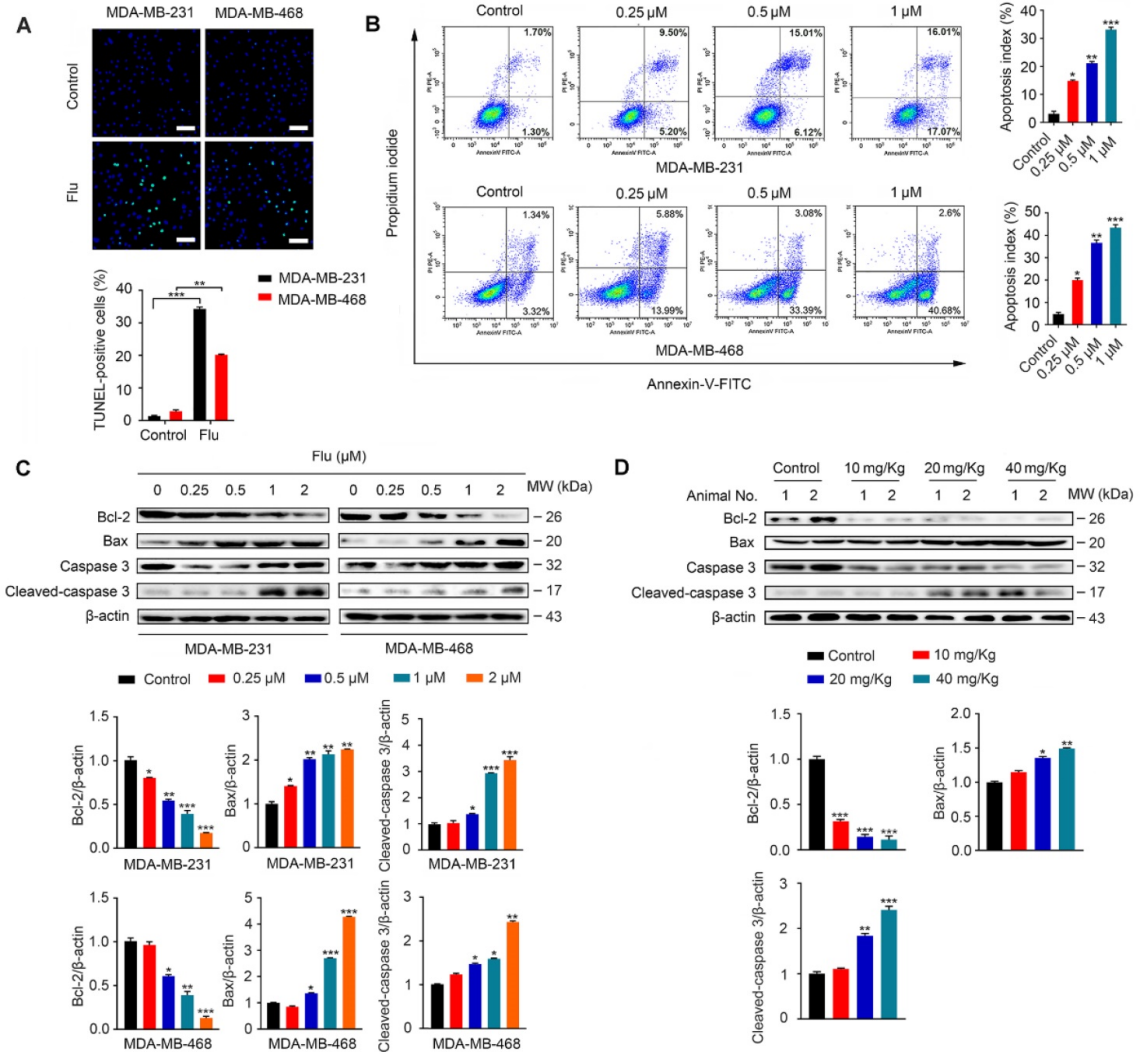
** Flubendazole induces apoptosis in TNBC cells.** (**A**) TUNEL assay in MDA-MB-231 and MDA-MB-468 cells treated with or without flubendazole (0.5 µM) for 24 h. Representative images and quantification of TUNEL-positive cells were shown. Scale bar, 50 µm. (**B**) MDA-MB-231 and MDA-MB-468 cells were treated with indicated concentrations of flubendazole for 24 h, apoptosis ratios were determined by flow cytometry analysis of Annexin-V/PI double staining. Representative images and quantification of apoptosis were shown. (**C**) Immunoblotting analysis of Bcl-2, Bax, caspase 3, as well as cleaved-caspase 3 expression in MDA-MB-231 and MDA-MB-468 cells treated with the indicated concentrations of flubendazole for 24 h. β-actin was used as a loading control. Quantification of immunoblotting analysis were shown. (**D**) Immunoblotting analysis of MDA-MB-231 xenograft tumor tissues from vehicle or flubendazole (20 mg/Kg) treated nude mice for expression of Bcl-2, Bax, caspase 3 and cleaved-caspase 3. β-actin was used as a loading control. Quantification of immunoblotting analysis were shown. Data are expressed as mean ± SEM. All data were representative of at least three independent experiments. *, *P* < 0.05, **, *P* < 0.01, ***, *P* < 0.001. Statistical significance compared with respective control groups.

It has been previously shown that flubendazole can induce apoptosis in multiple types of cancer 16-18. To examine whether apoptosis was associated with the antineoplastic effect of flubendazole in TNBC, we evaluated the apoptotic ratio *in vitro* using TUNEL assays. As a result, we verified that flubendazole could increase the TUNEL-positive ratio in MDA-MB-231 and MDA-MB-468 breast cancer cells (Figure 2A). We further performed flow cytometry analysis with Annexin-V/PI to confirm that flubendazole could induce TNBC cell death (Figure 2B). Subsequently, we checked the expression of apoptosis-related proteins *in vitro* (Figure 2C). Upon high concentrations of flubendazole, increased cleavage of caspase 3 was observed in both MDA-MB-231 and MDA-MB-468 cells. Likewise, flubendazole led to the downregulation of Bcl-2 and enhanced the expression of Bax. To confirm whether flubendazole could also induce apoptosis *in vivo*, we carried out western blot analysis of tumor tissues from vehicle or flubendazole- treated nude mouse (Figure 2D). As expected, results indicated that flubendazole could also induce apoptosis *in vivo*.

### Flubendazole induces autophagic cell death in TNBC cells

**Figure 3:**
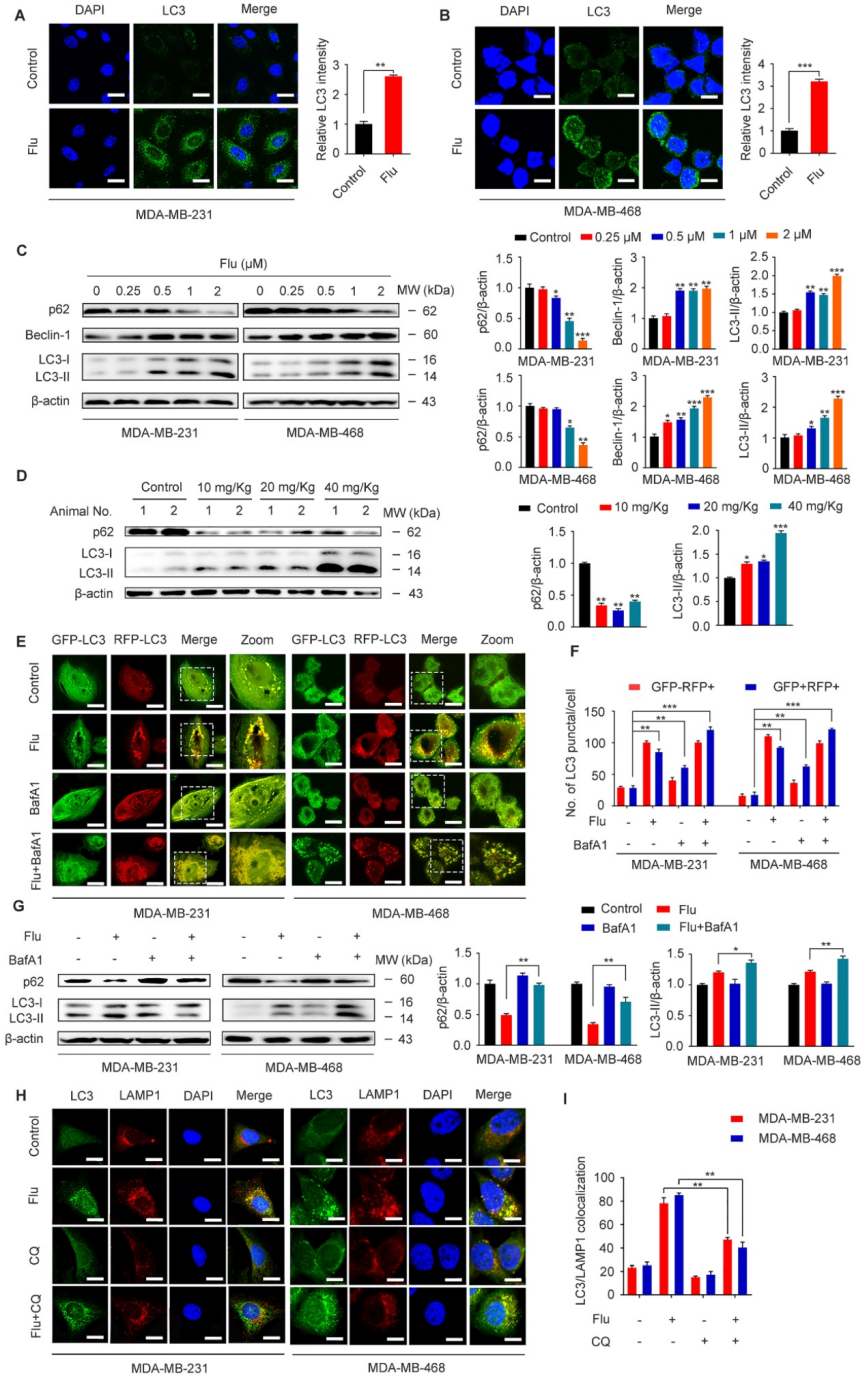
**Flubendazole induces autophagy in TNBC cells.** (**A-B**) Immunofluorescence analysis of the endogenous LC3B puncta in MDA-MB-231 and MDA-MB-468 cells treated with or without flubendazole (0.5 µM) for 24 h. Representative images with quantification of LC3 intensity were shown. Scale bar, 20 µm. (**C**) Immunoblotting analysis of p62, Beclin-1, LC3 expression in MDA-MB-231 and MDA-MB-468 cells treated with the indicated concentrations of flubendazole for 24 h. β-actin was used as a loading control. Quantification of immunoblotting analysis were shown. (**D**) Immunoblotting analysis of MDA-MB-231 xenograft tumor tissues from control or flubendazole (20 mg/Kg) treated nude mice for expression of p62 and LC3. β-actin was used as a loading control. Quantification of immunoblotting were shown. (**E-F**) MDA-MB-231 and MDA-MB-468 cells were transfected with GFP/mRFP-LC3 plasmid, after co-incubation with flubendazole (0.5 µM) in the presence or absence of BafA1 (10 nM). Representative images and quantitative analysis of LC3 puncta were shown. Scale bar, 10 µm. (**G**) MDA-MB-231 and MDA-MB-468 cells were co-incubated with flubendazole (0.5 µM) in the presence or absence of BafA1 (10 nM), then the expression levels of p62 and LC3 were detected. β-actin was measured as loading control. Quantification of immunoblotting analysis were shown. (**H**) Immunofluorescence analysis of the colocalization of endogenous LC3 with LAMP1 after treatment of flubendazole (0.5 µM) with or without CQ (10 mM) for 24 h in MDA-MB-231 and MDA-MB-468 cells. Scale bar, 10 µm. (**I**) The number of colocalized or non-colocalized LC3 and LAMP1 was quantified. Data are expressed as mean ± SEM. All data were representative of at least three independent experiments. *, *P* < 0.05, **, *P* < 0.01, ***, *P* < 0.001. Statistical significance compared with respective control groups.

Previous studies have shown that flubendazole is a potent inducer of autophagy initiation and flux in human cervical carcinoma HeLa cells 29. Therefore, we verified whether flubendazole could similarly induce autophagy in TNBC. Accordingly, we found a significant increase in the IF intensity of LC3 after flubendazole treatment *in vitro* (Figure 3A-B). Consistent with previous reports, we observed that flubendazole was able to induce a remarkable up-regulation of Beclin-1 and the degradation of p62, as well the conversion of LC3-I to LC3-II in a dose-dependent manner (Figure 3C). Meanwhile, we found that the positive rate of p62 was predominantly diminished (Figure 3D, Figure S5A and Figure S3C), and LC3 significantly accumulated (Figure 3D, Figure S5B and Figure S3B) in flubendazole-treated tumor tissues. Taken together, these data reveal that autophagy might be induced by flubendazole in TNBC cells.

Increased LC3-II levels can be associated with either enhanced autophagosome synthesis or autophagosome turnover. Hence, in order to further clarify the role of flubendazole in autophagy, MDA-MB-231 and MDA-MB-468 cells were first transfected with GFP/mRFP-LC3 expression vector and the formation of fluorescent autophagosomes (in yellow) and autolysosomes (in red) was further examined. Co-treatment with bafilomycin A1 (BafA1) and flubendazole led to further accumulation of autophagosomes, suggesting that flubendazole-induced autophagy is a continual process (Figure 3E-F). We have also detected increased accumulation of p62 and LC3 upon BafA1 treatment, suggesting that flubendazole may induce autophagic flux in TNBC (Figure 3G). To detect the formation of autophagic lysosomes, we evaluated the putative co-localization of endogenous LC3 puncta with LAMP1. Similarly, a clear co-localization of these two endogenous proteins was found in flubendazole-treated TNBC cells. Conversely, chloroquine was able to prevent this co-localization process (Figure 3H-I). These results support the notion that flubendazole may activate a complete autophagic flux in TNBC cells.

**Figure 4:**
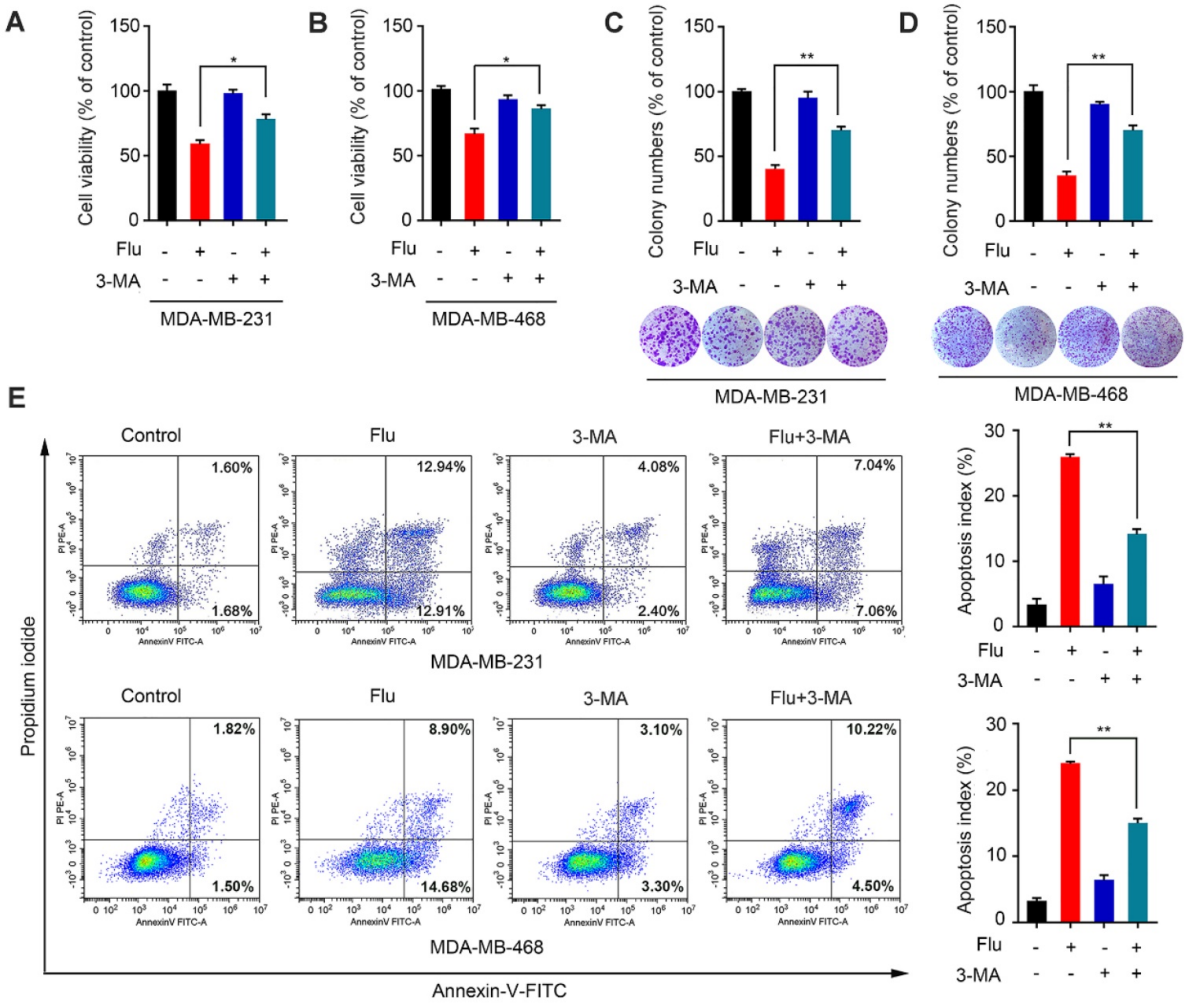
** Flubendazole induces autophagic cell death in TNBC cells.** (**A-B**) MDA-MB-231 and MDA-MB-468 cells were treated with flubendazole (0.5 µM) alone or in combination with 3-MA (1 mM) for 24 h, 3-MA was added 1 h before treatment of flubendazole. After treatment, cell viability was measured by MTT assay. (**C-D**) Colony formation assay of MDA-MB-231 and MDA-MB-468 cells treated with flubendazole (0.5 µM) alone or in combination with 3-MA (1 mM). Representative images and quantification of colonies were shown. (**E**) MDA-MB-231 and MDA-MB-468 cells were treated with flubendazole (0.5 µM) alone or in combination with 3-MA (1 mM) for 24 h, and apoptosis ratios were determined by flow cytometry analysis of Annexin-V/PI double staining. 3-MA was added 1 h before treatment of flubendazole. Representative images and quantification of apoptosis were shown. Data are expressed as mean ± SEM. All data were representative of at least three independent experiments. *, *P* < 0.05, **, *P* < 0.01, ***, *P* < 0.001. Statistical significance compared with respective control groups.

Given that autophagy acts as a “double-edged sword” during cancer progression 28-29, 31-34, we further explored the relationship between flubendazole-mediated autophagy and growth inhibition of TNBC cells. The autophagy inhibitor 3-methyladenine (3-MA), which is capable of inhibiting autophagy by interrupting autophagosome formation, was employed to block autophagy induction. Thus, we found that viability of cells co-treated with flubendazole and 3-MA was significantly higher than those treated with flubendazole only (Figure 4A-B). This result was further confirmed by clonogenic assays (Figure 4C-D), reiterating that the inhibition of TNBC cell growth due to flubendazole treatment was autophagy-dependent. Thereafter, we performed flow cytometry analysis to examine the apoptosis rate of TNBC cells co-treated with flubendazole and 3-MA (Figure 4E). Respective results showed that 3-MA could partially reverse the apoptosis induced by flubendazole. Altogether, these data suggest that flubendazole can induce autophagic cell death in TNBC cells.

### Flubendazole exerts anti-migration potential through autophagy of TNBC cells

**Figure 5:**
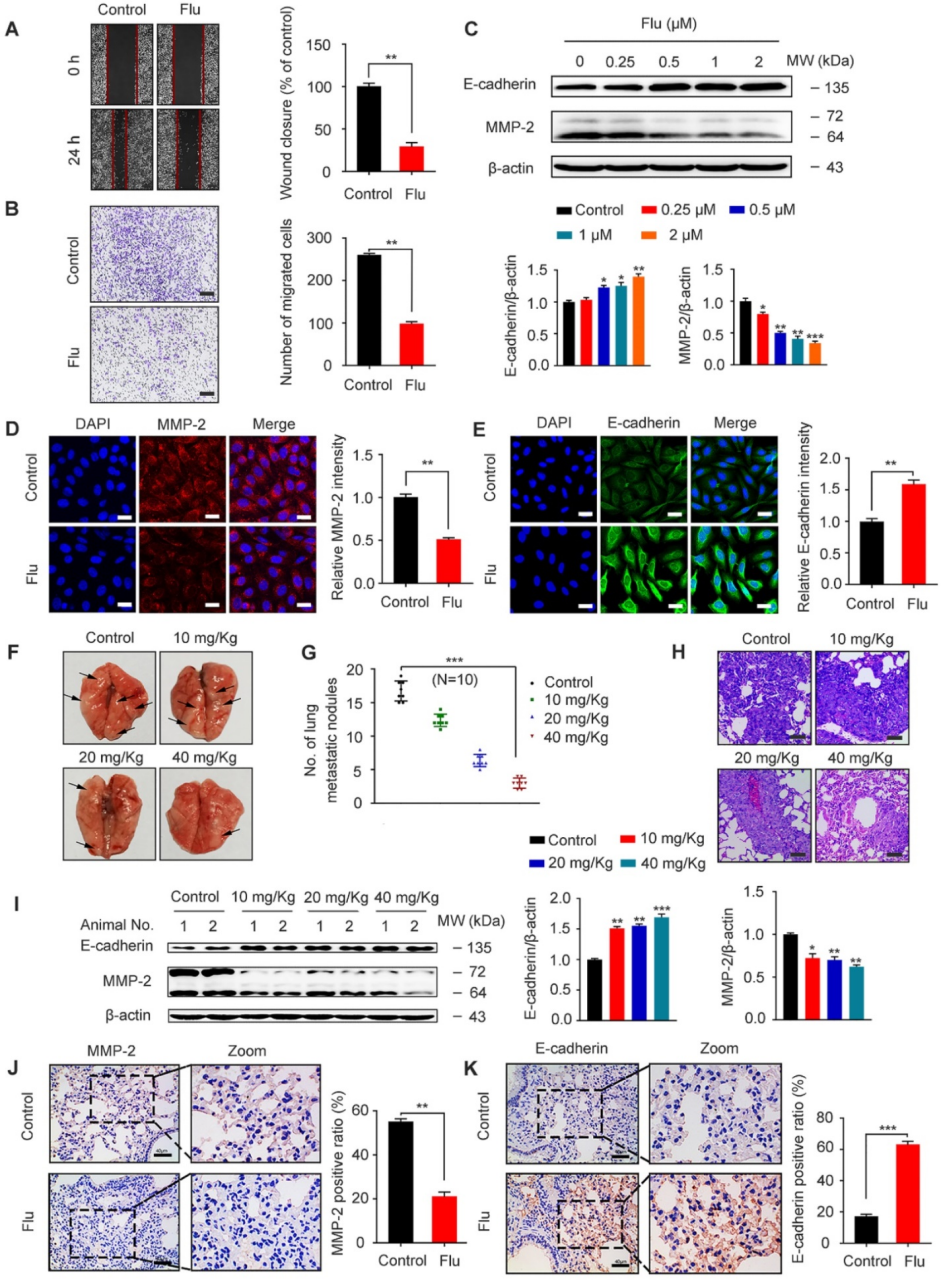
** Flubendazole exerts anti-migration potential *in vivo* and *in vitro*.** (**A-B**) MDA-MB-231 cells were treated with or without flubendazole (0.5 µM). The scratch assay and transwell assay were used to measure migration capabilities of the cells. The wound closure ratio represents the level of cell migration ability. Representative images and statistics were shown. Scale bar, 100 µm. (**C**) Immunoblotting analysis of MMP-2 and E-cadherin expression in MDA-MB-231 cells treated with the indicated concentrations of flubendazole for 24 h. β-actin was measured as a loading control. Quantification of immunoblotting analysis were shown. (**D-E**) Immunofluorescence analysis of MMP-2 and E-cadherin levels in MDA-MB-231 cells treated with or without flubendazole (0.5 µM) for 24 h. Quantification of immunofluorescence analysis were shown. Scale bar, 20 µm. (**F**) Representative images of lung metastasis from control or flubendazole treated nude mice, the MDA-MB-231 cells were injected into the vein of mice. The arrow represents pulmonary metastatic nodule. (**G**) Tumor cell metastasis was examined by counting metastatic nodules in mouse lung. (**H**) Representative H&E staining of lung sections from control or flubendazole treated nude mice (n = 10). Scale bar, 40 µm. (**I**) Immunoblotting analysis of MMP-2 and E-cadherin expression in lung tissues from control or flubendazole (20 mg/Kg) treated nude mice. β-actin was measured as a loading control. Quantification of immunoblotting were shown. (**J-K**) The expression of MMP-2 and E-cadherin in representative lung sections of nude mice from control and median dose (20 mg/Kg). Representative images and quantitative analysis of the percentage of positive ratios were shown. Scale bar, 40 µm. Data are expressed as mean ± SEM. All data were representative of at least three independent experiments. *, *P* < 0.05, **, *P* < 0.01, ***, *P* < 0.001. Statistical significance compared with respective control groups.

TNBC patients often have a poor disease prognosis due to the metastasis, which mainly occurs in the lungs 6, 35. We further analyzed the effect of flubendazole on MDA-MB-231 cell migration by scratch assay and as a result, we found that wound closure ratio was decreased in cells treated with flubendazole (Figure 5A). Moreover, transwell assays were used to confirm that flubendazole could eventually inhibit the migration of MDA-MB-231 cells (Figure 5B). According to western blotting and IF analyses, we also observed an up-regulation of E-cadherin and down-regulation of MMP-2 in MDA-MB-231 cells, in response to flubendazole treatment (Figure 5C-E).

In order to reveal the efficacy of flubendazole *in vivo*, we established a xenograft tumor model by injecting MDA-MB-231 cells into the tail vein of nude mice. Cell metastatic was examined by counting the number of pulmonary metastatic nodules (Figure 5F-G) and then analyzing the respective lung sections by H&E staining (Figure 5H). As indicated, flubendazole treatment *in vivo* was able to decrease lung metastatic nodules. Furthermore, we observed that the protein levels of MMP-2 significantly decreased while E-cadherin levels increased in the lung tissue of flubendazole-treated mice (Figure 5I-K and Figure S3D-E). These results indicate that flubendazole has an anti-migration activity towards TNBC *in vitro* and* in vivo*.

**Figure 6:**
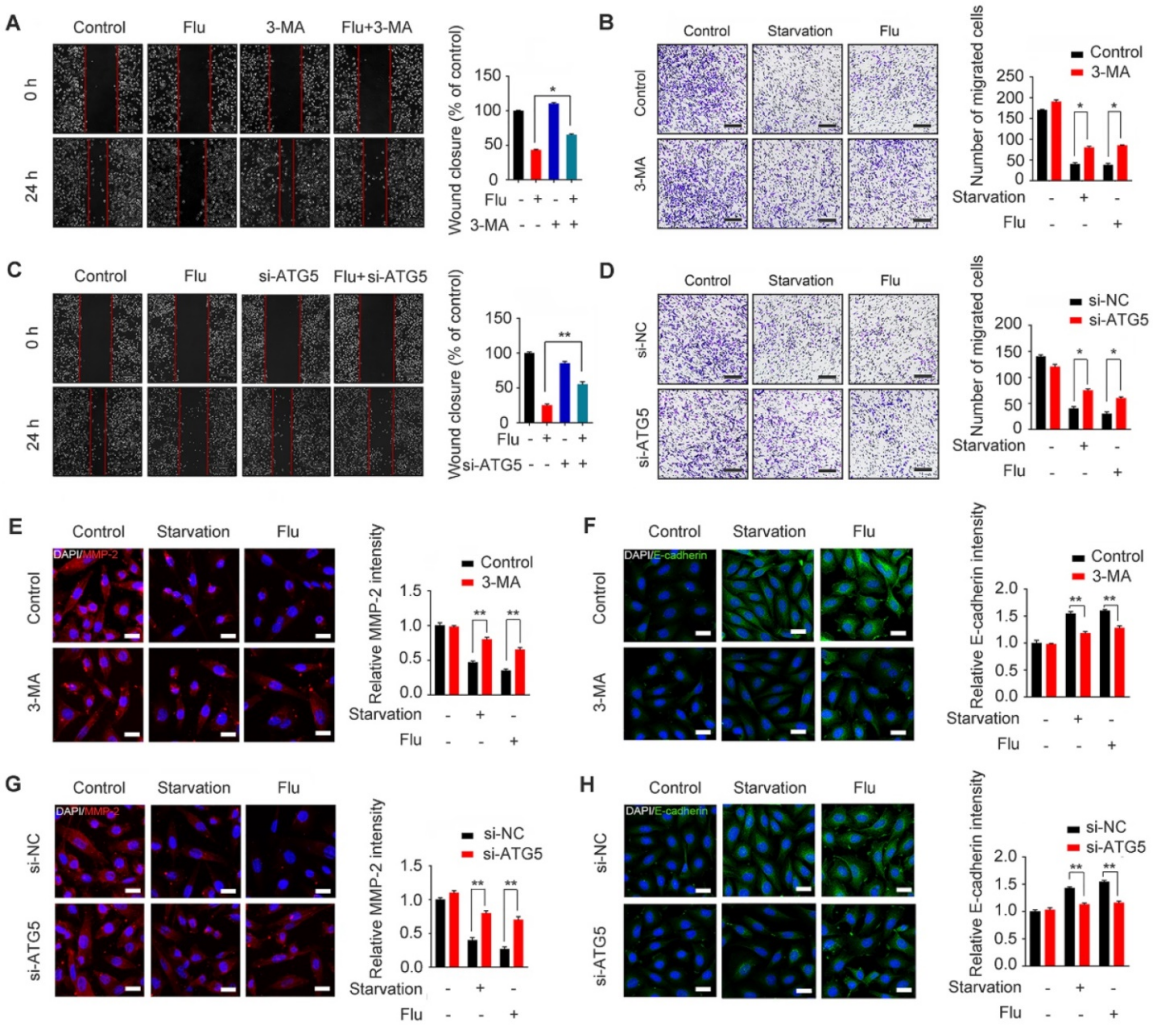
Flubendazole inhibits MDA-MB-231 cells migration through autophagy *in vitro*. (**A-B**) MDA-MB-231 cells were treated with flubendazole (0.5 µM) alone or in combination with 3-MA (1 mM) for 24 h, 3-MA was added 1 h before treatment of flubendazole. Then scratch assay and transwell assay were used to measure the migration capabilities of the cells. Representative images and statistics were shown. Scale bar, 100 µm. (**C-D**) MDA-MB-231 cells were transfected with control or ATG5-siRNA, followed by treatment with or without flubendazole (0.5 µM) for 24 h. Then scratch assay and transwell assay were used to measure the migration capabilities of the cells. Representative images and statistics were shown. Scale bar, 100 µm. (**E-F**) MDA-MB-231 cells were treated with flubendazole (0.5 µM) alone or in combination with 3-MA (1 mM) for 24 h, 3-MA was added 1 h before treatment of flubendazole. The expression of MMP-2 and E-cadherin were analyzed by immunofluorescence. Scale bar, 20 µm. (**G-H**) MDA-MB-231 cells were transfected with control or ATG5-siRNA, followed by treatment with or without flubendazole (0.5 µM) for 24 h. The expression of MMP-2 and E-cadherin were analyzed by immunofluorescence. Scale bar, 20 µm. Data are expressed as mean ± SEM. All data were representative of at least three independent experiments. *, *P* < 0.05, **, *P* < 0.01, ***, *P* < 0.001. Statistical significance compared with respective control groups.

To gain insights into the mechanism by which flubendazole inhibits TNBC migration, we further assessed the association between migration and autophagy in flubendazole-treated MDA-MB-231 cells. For this, we first combined flubendazole treatment with 3-MA or ATG5 silencing (Figure S6), as indicated, we found that inhibiting autophagy *in vitro* was able to increase the wound closure ratio and the number of migrating MDA-MB-231 cells (Figure 6A-D). The ability of flubendazole to down-regulate MMP-2 and up-regulate E-cadherin was also attenuated by 3-MA treatment or ATG5 knockdown (Figure 6E-H), suggesting that autophagy is a key link in the inhibition of TNBC migration by flubendazole. Thus, flubendazole exerts an anti-migration role in TNBC progression through autophagy both *in vitro* and *in vivo.*

### Identification of flubendazole-induced autophagy-associated gene alteration by RNA-seq analysis

**Figure 7:**
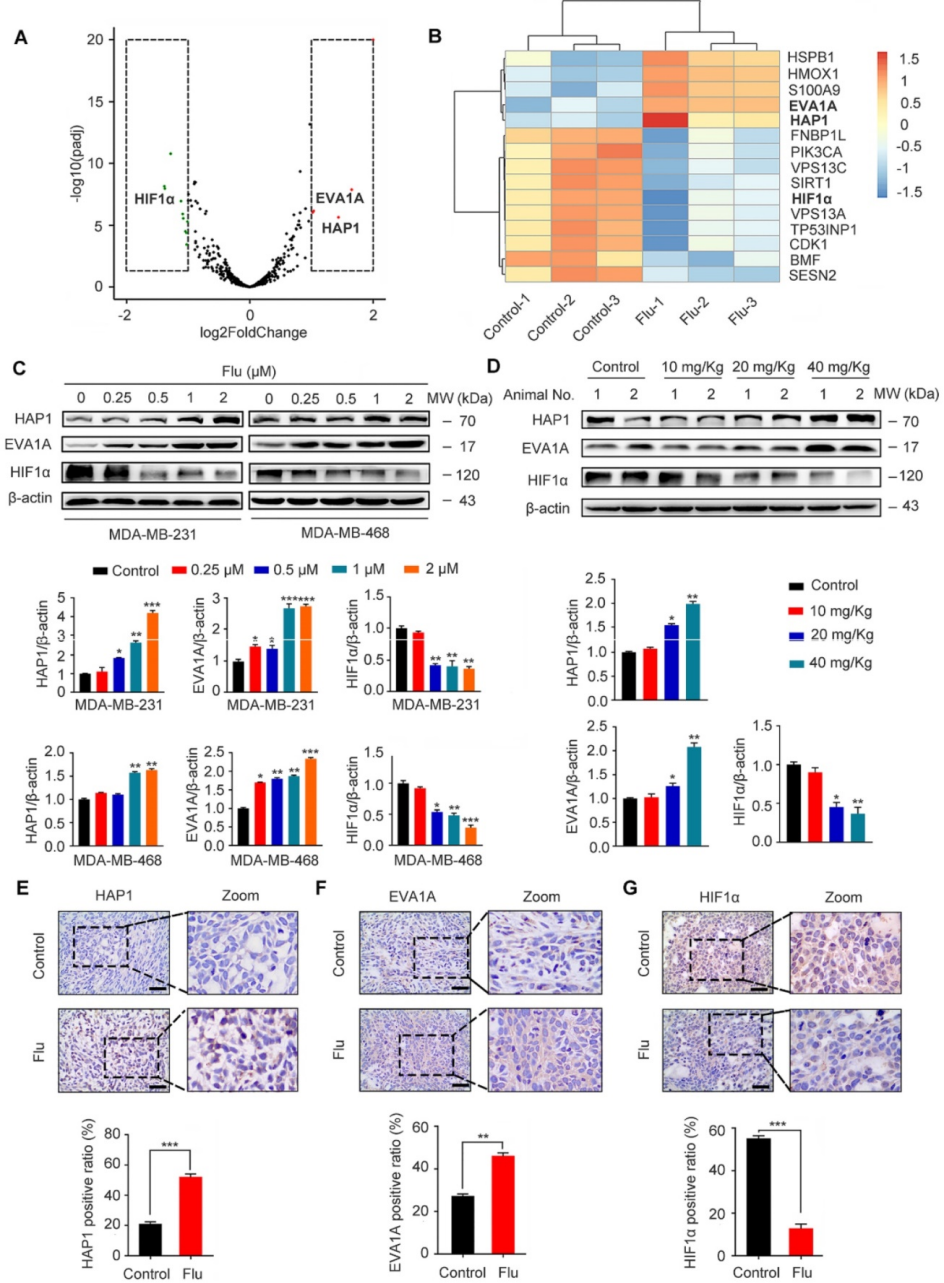
Flubendazole regulates the expression of HAP1, EVA1A and HIF1α *in vitro* and *in vivo*. (**A**) Volcano plot of autophagy-related genes with significant differences expression in MDA-MB-231 cells treated with or without flubendazole (0.5 µM). Red plots represent padj < 0.05, log2FoldChange > 1 and green plots represent padj < 0.05, log2FoldChange < -1. (**B**) Heatmap of autophagy-related genes with significant differences expression in MDA-MB-231 cells treated with or without flubendazole (0.5 µM). (**C**) Immunoblotting analysis of HAP1, EVA1A and HIF1α expression in MDA-MB-231 cells treated with the indicated concentrations of flubendazole for 24 h. β-actin was measured as a loading control. Quantification of immunoblotting analysis were shown. (**D**) Immunoblotting analysis of HAP1, EVA1A and HIF1α expression in MDA-MB-231 xenograft tumor sections from control or flubendazole (20 mg/Kg) treated nude mice. β-actin was measured as a loading control. Quantification of immunoblotting were shown. (**E-G**) The expression of HAP1, EVA1A and HIF1α in representative MDA-MB-231 xenograft tumor sections of nude mice from vehicle and median dose (20 mg/Kg). Representative images and quantitative analysis of the percentage of positive ratios were shown. Scale bar, 40 µm. Data are expressed as mean ± SEM. All data were representative of at least three independent experiments. *, *P* < 0.05, **, *P* < 0.01, ***, *P* < 0.001. Statistical significance compared with respective control groups.

To uncover the potential mechanism(s) of flubendazole-induced autophagy, we further treated MDA-MB-231 cells with flubendazole for 24 h and then performed RNA-seq analysis to examine the content of differentially expressed genes. A total of 529 genes (261 and 268 up- and downregulated genes, respectively) were differentially expressed in flubendazole-treated samples when compared with the control (Table S1). We screened out autophagy-related genes (i.e. 5 up- and 10 down-regulated genes; absolute log2 (fold change) > 1), detected with a significant difference in expression (Figure 7A-B). Among these genes, it has been reported that EVA1A and HAP1 expression is decreased in tumor tissues, while HIF1α is dramatically increased 36-38. Interestingly, RNA-seq analysis showed that flubendazole could reverse these molecular events by decreasing, for instance, HIF1α levels when compared with the control group (Figure 7C-D, Figure 7G, Figure S3H, Figure S7A and Figure S8C). Moreover, the expression of EVA1A and HAP1 was up-regulated upon flubendazole treatment in a dose-dependent manner, both *in vitro* and *in vivo* (Figure 7C-F, Figure S3F-G and Figure S8A-B).

### Flubendazole induces autophagy and exerts anti-proliferative effects by targeting EVA1A in TNBC cells

**Figure 8:**
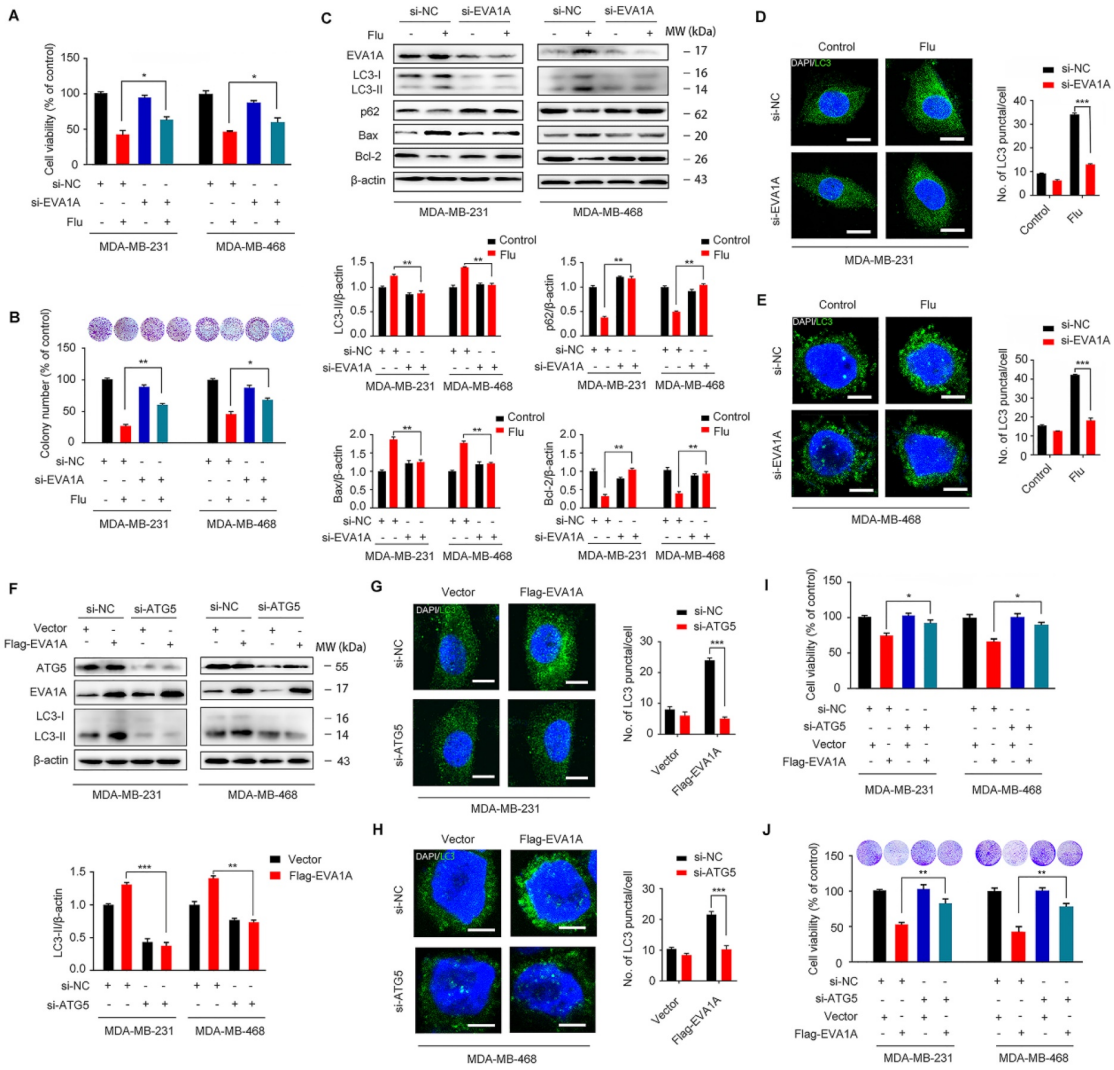
Flubendazole elicits anti-cancer effects via targeting EVA1A-modulated autophagy and apoptosis in TNBC cells. (**A**) MDA-MB-231 and MDA-MB-468 cells were transfected with negative-control or EVA1A siRNA, respectively. After treatment with or without flubendazole (0.5 µM) for 24 h, cell viability was measured by MTT assay. (**B**) MDA-MB-231 and MDA-MB-468 cells were transfected with negative-control or EVA1A siRNA, respectively. After treatment with or without flubendazole (0.5 M) for 2 weeks. Representative images and quantification of colonies were shown. (**C**) MDA-MB-231 and MDA-MB-468 cells were transfected with negative-control or EVA1A siRNA, followed by treatment with or without flubendazole (0.5 µM) for 24 h. Then, the expression levels of EVA1A, p62, LC3, Bax and Bcl-2 were determined by immunoblotting analysis. β-actin was measured as a loading control. (**D-E**) MDA-MB-231 and MDA-MB-468 cells were transfected with negative-control or EVA1A siRNA, followed by treatment with or without flubendazole (0.5 µM) for 24 h. Representative images with quantification of LC3 intensity were shown. Scale bar, 10 µm. (**F**) MDA-MB-231 and MDA-MB-468 cells were co-transfected with EVA1A siRNA and Flag-EVA1A or vehicle control respectively for 24 h, followed by treatment with or without flubendazole (0.5 µM) for 24 h. Immunoblotting analysis of ATG5, EVA1A and LC3 expression. β-actin was measured as a loading control. Quantification of immunoblotting were shown. (**G-H**) MDA-MB-231 and MDA-MB-468 cells were co-transfected with EVA1A siRNA and Flag-EVA1A or vehicle control respectively for 24 h, followed by treatment with or without flubendazole (0.5 µM) for 24 h. Representative images with quantification of LC3 intensity were shown. Scale bar, 10 µm. (**I**) MDA-MB-231 and MDA-MB-468 cells were co-transfected with EVA1A siRNA and Flag-EVA1A or vehicle control respectively for 24 h, followed by treatment with or without flubendazole (0.5 µM) for 24 h, cell viability was measured by MTT assay. (**J**) MDA-MB-231 and MDA-MB-468 cells were co-transfected with EVA1A siRNA and Flag-EVA1A or vehicle control respectively for 24 h. After treatment with or without flubendazole (0.5 µM) for 2 weeks. Representative images and quantification of colonies were shown. Data are expressed as mean ± SEM. All data were representative of at least three independent experiments. *, *P* < 0.05, **, *P* < 0.01, ***, *P* < 0.001. Statistical significance compared with respective control groups.

EVA1A (Eva-1 homolog A), also known as TMEM166 (transmembrane protein 166) or FAM176A (sequence similarity 176 families), is a vacuole-located type I membrane protein, and its expression is typically diminished in a variety of tumor cells 39-45. In fact, it has been reported that EVA1A can induce cell death associated with autophagy and apoptosis 30, 46, so restoring EVA1A expression may promote tumor suppression 40, 42. We hypothesized that EVA1A could serve as a potential target for flubendazole-induced autophagic cell death *in vitro*. So, we first examined the role of EVA1A in flubendazole-mediated apoptosis and autophagy in TNBC cells. To explore whether flubendazole induced autophagic cell death by targeting EVA1A, the specific small-interfering RNAs were transfected into TNBC cells to silence EVA1A expression. As a result, we verified that EVA1A knockdown could significantly restore cell growth in flubendazole-treated TNBC cells (Figure 8A-B). We also observed that EVA1A gene silencing partially blocked LC3 puncta accumulation (Figure 8D-E), p62 degradation and LC3 lipidation in cells treated with flubendazole (Figure 8C), indicating that this drug can affect autophagy of TNBC cells by regulating EVA1A levels. Down-regulation of Bax levels and up-regulation of Bcl-2 indicated that the ability of flubendazole to induce apoptosis in TNBC was reduced upon silencing EVA1A expression (Figure 8C). Thereafter, we investigated the effect of EVA1A on TNBC cell migration after flubendazole treatment. Interestingly, we found that EVA1A-depleted TNBC cells treated with flubendazole had a higher migration capacity than flubendazole-treated wild-type (control) cells (Figure S9A). This observation was consistent with IF results for MMP-2 and E-cadherin (Figure S9B-C).

Accumulating evidence has revealed that EVA1A is a prominent tumor suppressor molecule. The restoration of EVA1A levels in some cancer cell lines is capable of inducing cell death by autophagy and apoptosis-related mechanisms, so inhibition of autophagy and apoptosis could reduce EVA1A-induced cell death 40-42. We have previously demonstrated that flubendazole can induce autophagic cell death in TNBC cells. Then, we further explored the effect of EVA1A overexpression on autophagy in Atg5-depleted TNBC cells. We found that overexpression of EVA1A does not induce the aggregation of LC3 spots in ATG5 knockdown MDA-MB-231 and MDA-MB-468 cells (Figure 8F-H), thus indicating that EVA1A-induced autophagy is ATG5-dependent. Next, we analyzed the effect of EVA1A overexpression on cell growth and proliferation in both normal and Atg5-depleted TNBC cells. We observed that EVA1A overexpression could significantly inhibit cell growth and proliferation in normal cells, but this inhibitory effect was attenuated in Atg5-depleted TNBC cells (Figure 8I-J). Therefore, EVA1A-mediated induction of autophagy appears necessary for flubendazole to inhibit TNBC cell growth and proliferation.

**Figure 9:**
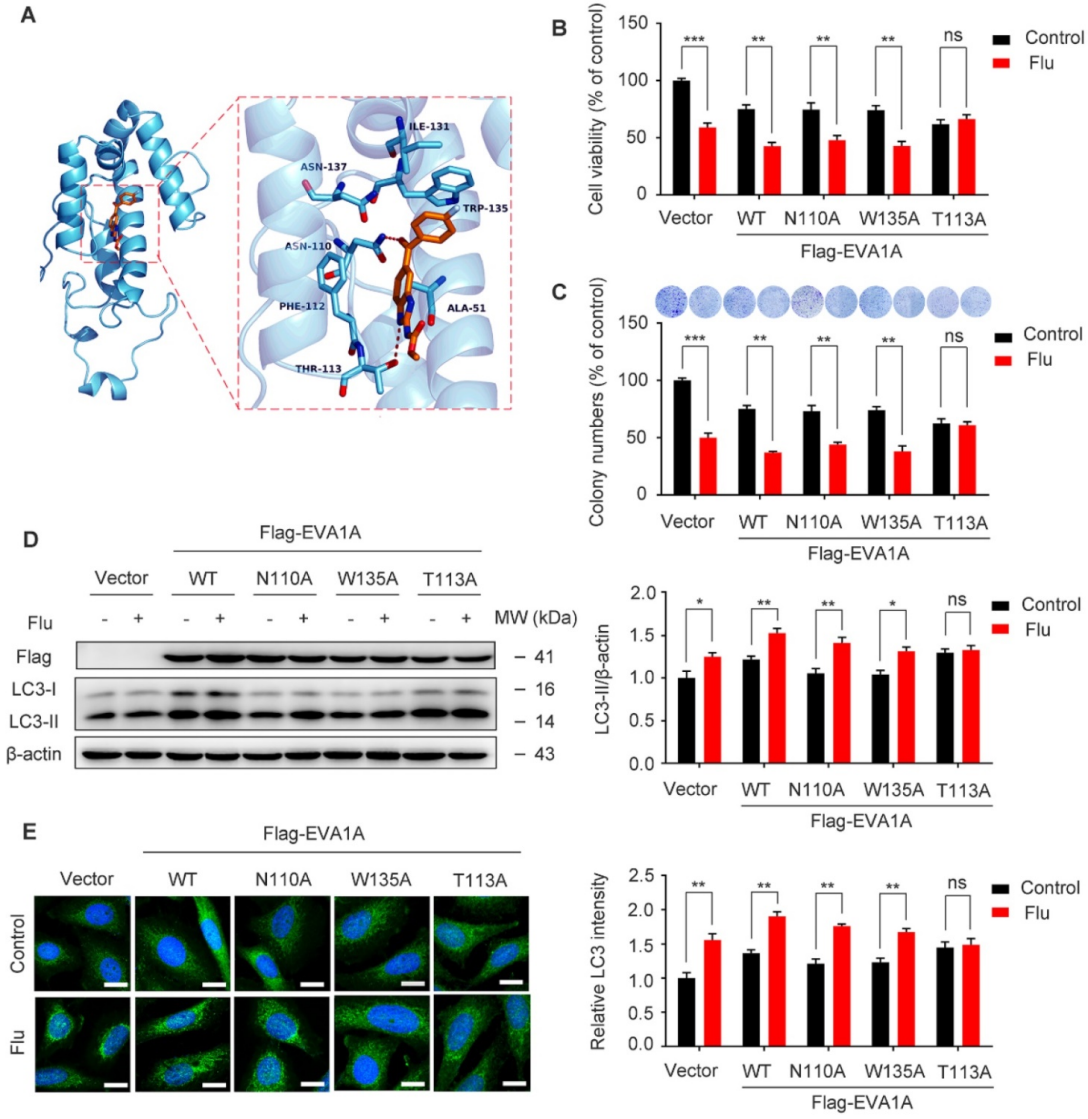
Effect of EVA1A mutant on the proliferation and autophagy regulated by flubendazole in TNBC cells. (**A**) The predicted binding mode of flubendazole with EVA1A. General and detailed views of interactions between flubendazole and EVA1A were presented. (**B**) MDA-MB-231 cells were transfected with empty vector, wild type EVA1A (EVA1A^WT^) and EVA1A mutants (EVA1A^N110A^, EVA1A^W135A^, EVA1A^T113A^) respectively for 24 h, followed by treatment with or without flubendazole (0.5 µM) for 24 h, cell viability was measured by MTT assay. (**C**) MDA-MB-231 cells were transfected with empty vector, wild type EVA1A (EVA1A^WT^) and EVA1A mutants (EVA1A^N110A^, EVA1A^W135A^, EVA1A^T113A^) respectively for 24 h. After treatment with or without flubendazole (0.5 µM) for 2 weeks. Representative images and quantification of colonies were shown. (**D**) MDA-MB-231 cells were transfected with empty vector, wild type EVA1A (EVA1A^WT^) and EVA1A mutants (EVA1A^N110A^, EVA1A^W135A^, EVA1A^T113A^) respectively for 24 h, followed by treatment with or without flubendazole (0.5 μM) for 24 h. Then, the expression levels of EVA1A and LC3 was determined by immunoblotting analysis. β-actin was measured as a loading control. (**E**) MDA-MB-231 cells were transfected with empty vector, wild type EVA1A (EVA1A^WT^) and EVA1A mutants (EVA1A^N110A^, EVA1A^W135A^, EVA1A^T113A^) respectively for 24 h, followed by treatment with or without flubendazole (0.5 µM) for 24 h. Representative images with quantification of LC3 intensity were shown. Scale bar, 20 µm. Data are expressed as mean ± SEM. All data were representative of at least three independent experiments. *, *P* < 0.05, **, *P* < 0.01, ***, *P* < 0.001. Statistical significance compared with respective control groups.

To confirm the binding mode of flubendazole with EVA1A, five structural models of EVA1A were predicted and the optimal docked conformation was obtained accordingly. We found that flubendazole could form a π-π interaction with the indole side chain of Trp135 as well as by hydrogen bonds with Thr113 and Asn110 (Figure 9A). We speculated that these three amino acid residues were part of the activation binding sites of flubendazole in EVA1A. Therefore, several EVA1A mutants (EVA1A^W135A^, EVA1A^T113A^, EVA1A^N110A^) were constructed by site-directed mutagenesis to study the effects of these point mutations on flubendazole-induced autophagy and cell death. Interestingly, the mutants EVA1A^T113A^ significantly attenuated the inhibitory effect of flubendazole in MDA-MB-231 cells when compared to the wild-type (WT) EVA1A, EVA1A^W135A^ and EVA1A^N110A^ mutants (Figure 9B). Consistently, clone formation experiments also indicated that the anti-proliferative effect of flubendazole was alleviated (Figure 9C). Moreover, EVA1A^T113A^ could significantly blocked LC3 puncta accumulation and LC3 lipidation (Figure 9D-E). These results indicate that Thr113 may be the key amino acid residues for the binding of flubendazole to EVA1A, and play an important role in regulating the proliferation and autophagy of TNBC cells.

## Discussion

For decades, flubendazole has been widely used to treat human infections against worm and intestinal parasites. In tumor cells, flubendazole has been recently shown to affect microtubule dynamics, inhibit angiogenesis and cell metastasis, as well as to induce mitotic catastrophe and apoptosis 15-24. Mechanistically, flubendazole may also target NF-κB signaling, which is required for esophageal squamous carcinoma cell survival 17. In colorectal and breast cancer cells, flubendazole exerts anti-tumor and anti-metastatic activities by inhibiting STAT3 22, 26.

As a highly regulated catabolic progress, autophagy plays a dual role in the occurrence and development of cancer 47. Depending on the context, it may positively or negatively influence cancer metastasis 48, which is ultimately the primary cause of cancer-associated mortality. A number of studies have indicated a close relationship between flubendazole and autophagy, though the exact mechanism has been not characterized, especially in the context of TNBC 22, 28.

In this study, we have elucidated flubendazole's anti-cancer effect and underlying mechanisms in TNBC. For this, we showed a remarkably inhibitory potential of the drug towards the growth and migration of TNBC cells both *in vitro* and *in vivo*. However, due to the limited cell lines we use, it may be necessary to extend the studies over other breast cancer cell lines or primary cultures derived from patients to make the conclusion more general. We have also validated the occurrence of apoptosis and autophagy upon flubendazole treatment. At the same time, the combinatorial treatment of flubendazole with autophagy inhibitors or knockdown of ATG5 was able to suppress the anti-proliferative and anti-migration activity of flubendazole in TNBC cells. Additionally, we have discovered that flubendazole may up-regulate the expression of EVA1A, a gene which is known to play major roles in autophagy and apoptosis. Indeed, EVA1A appears to the key component behind flubendazole in governing proliferation and migration of TNBC cells.

Due to its essential role in cancer, autophagy has become a potential target for cancer therapy 14. Numerous small molecules have been found to target autophagy, and possibly be applied in TNBC treatment. SLLN-15, a novel orally available seleno-purine molecule, can suppress TNBC cell proliferation and progression to metastasis by inducing cytostatic autophagy 31. Previous reports have shown that FL-411, a small-molecule inhibitor of BRD4, can induce BRD4-AMPK-modulated autophagy/autophagic cell death in breast cancer 32. LYN-1604, an agonist of ULK1, has a good therapeutic potential in TNBC by targeting ULK1-modulated cell death 33-34. In this work, *in vitro* and *in vivo* data showed that the therapeutic potential of flubendazole could be reduced when combined with 3-MA or ATG5 depletion (known to suppress autophagy activity), thus implicating that autophagy may regulate the proliferation and migration of TNBC cells.

According to our RNA-seq analysis, flubendazole was able to significantly up-regulate EVA1A expression in MDA-MB-231 cells. Previous studies have revealed that, compared to normal tissues, EVA1A is down-regulated in various human tumors, such as pituitary adenoma, myeloma, adrenocortical, pancreatic and hepatocellular carcinomas, as well as gastric, esophageal and lung cancers 39-45. Restoring EVA1A expression may induce cell death by autophagy and apoptosis-related mechanisms 40, 42. Hence, we further confirmed that flubendazole could enhance the expression of EVA1A in TNBC cell lines and xenograft tumors. Upon silencing of EVA1A expression, the ability of flubendazole to induce autophagy and apoptosis was weakened in TNBC cells, while the tumor survival rate and MMP-2 levels were elevated. Furthermore, the anti-proliferation ability of flubendazole was decreased when ATG5 was depleted in EVA1A-overexpressing TNBC cells. In brief, flubendazole can regulate autophagy and apoptosis by targeting EVA1A, further affecting tumor proliferation and migration. Thereafter, we further identified that Thr113 may be the key amino acid residues involved in the binding of flubendazole to EVA1A, thus playing an important role in flubendazole-mediated TNBC suppression. However, our findings also exist some limitations, since the three sites were identified by homology modeling of the interaction between flubendazole and EVA1A, which cannot be confirmed as the accurate critical sites. Thus, our finding may need to be further validated after the resolution of the crystal structure of EVA1A.

Interestingly, some reports have also indicated that EVA1A is associated with autophagosome membrane development 30. Considering that flubendazole may activate Atg4B, a crucial component involved in Atg8/LC3 conjugation during autophagosome formation 28, we believe that the impact of EVA1A in Atg4B activation may deserve better clarification. Therefore, we look forward to characterizing the link (if any) between EVA1A and Atg4B, and whether flubendazole could regulate both of these factors to further affect autophagy in cancer.

## Conclusion

Here we report, for the first time, that flubendazole induces autophagic cell death and regulates autophagy and apoptosis by targeting EVA1A, which has an anti-proliferation and anti-migration value in TNBC. These results provide novel insights into exploring the anti-cancer efficacy of flubendazole, implying that this original anthelmintic drug may be repurposed as a novel anti-tumor agent. This study sheds light on the putative use of this new autophagy inducer in future TNBC therapeutics.

## Materials and Methods

### Cell culture and reagents

Cells were purchased from American Type Culture Collection (ATCC, Manassas, VA, USA). The U937, HL60 and MCF10A cell lines were cultured in Roswell Park Memorial Institute medium (RPMI) 1640 medium and the U87, SW480, RKO, LST174t, HCT116, HepG2, HUH7, SK-HepG1, A549, 786-0, Hela, MCF-7, MDA-MB-231 and MDA-MB-468 were maintained in Dulbecco's Modified Eagle Medium (DMEM) containing 10% fetal bovine serum (FBS) and 1% penicillin-streptomycin (Life Technologies) in 5% CO_2_ at 37 °C. Cells were grown to 70-80% confluence in cell culture dishes or plates and all the experiments were performed on logarithmically growing cells.

Flubendazole (SML2510), 3-(4,5-dimethyl-2-thiazolyl)-2,5-diphenyl-2-H-tetrazolium bromide (M2128), 3-MA (M9281), CQ (C6628), DAPI (D9542) were purchased from Sigma-Aldrich (St. Louis, MO, USA). Bafilomycin A1 (ab120497) and was purchased from Abcam (Cambridge, UK). Antibodies used in this study were as follow: Beclin1 (3495, CST), SQSTM1/p62 (8025, CST), LC3B (51520, Abcam), Bax (5023, CST), Bcl-2 (2870, CST), Caspase3 (9662, CST), MMP-2 (87809, CST), E-cadherin (14472, CST), HAP1(58600, Abcam), EVE1A (216043, Abcam), HIF1α (36169, CST), β-actin (66009-1-Ig, Proteintech, IL, USA), LAMP1(25630, Abcam), ATG5 (12994, Abcam), Anti-DDDDK tag (Binds to FLAG® tag sequence) (1162, Abcam).

### Cell viability assay

The cells were plated in 96-well plates at a density of 6 × 10^4^ cells/mL. After incubation at 37 °C for 24 h, cells were treated with different concentrations of flubendazole for the indicated time periods. Cell viability was measured by MTT assay.

### TUNEL assays

The One Step TUNEL Apoptosis Assay Kit (Beyotime) was used to detect apoptotic cells according to the manufacturer's instructions. The apoptotic and non-apoptotic signals were recorded using a fluorescent microscope and the percentage of cells with DNA nick end-labeling was evaluated.

### Lactate dehydrogenase (LDH) assay

Lactate dehydrogenase release was used to detect cytotoxicity following different treatments, using a lactate dehydrogenase (LDH) test kit (Beyotime, Nanjing, China). Studies were performed according to the manufacturer's instructions.

### GFP/mRFP - LC3 transfection

Cells were seeded into 24-well culture plates (2.5×10^4^ cells/well^)^. After incubation of 24 h, cells were transfected with GFP/mRFP-LC3 (HB-AP2100001, HANBIO, China) for 6 h. Then the transfected cells were used for subsequent experiments 36 h later and were analyzed under a fluorescence microscope.

### Immunofluorescence (IF) analysis

Cells were seeded onto the glass cover slips in 24-well plates. After treatment, cells were fixed with 4% paraformaldehyde in PBS for 30 min. The slides were then washed three times with PBS and incubated with 0.2% Triton X-100 (Sigma-Aldrich, 9002-93-1) and 5% goat serum (Sigma-Aldrich, G9023) for 30 min. Cells were incubated with indicated primary antibody overnight at 4°C and subsequently incubated with secondary antibody (TRITC, ab6718; FITC, ab6717) at room temperature for 1 h. Nuclei were finally stained with DAPI for 5 min. Images were captured using a confocal laser canning microscopy (Zeiss).

### Immunohistochemistry (IHC) analysis

Sections of the tumor and lung were submerged in EDTA antigenic retrieval buffer (pH 8.0) or citrate buffer (pH 6.0), and microwaved for antigenic retrieval. The slides were then incubated at 37℃ for 30-40 minutes with Ki67 antibody (1:400), p62 antibody (1:400), LC3 antibody (1:400), MMP-2 antibody (1:400) E-cadherin antibody (1:400), EVA1A antibody (1:200), HAP1 antibody (1:300) and HIF1α antibody (1:300), respectively. Normal rabbit/mouse IgG was used as a negative control. The slides were then treated by HRP polymer conjugated secondary antibody for 30 min and developed with diaminobenzidine solution. Meyer's hematoxylin was used as a counterstain.

### Immunoblotting (IB) analysis

All cells and animal tumors as well as lung tissues were collected and lysed by lysis buffer at 4 °C for 30 min. After 12000 rpm centrifugation for 10 min, the protein level of the supernatant was quantified by Bio-Rad DC protein assay (Bio-Rad Laboratories, Hercules, CA, USA). Equal amounts of the total protein were separated by 12% SDS-PAGE and electrophoretically transferred to PVDF membranes. Subsequently, membranes were blocked with 5% nonfat dried milk. Proteins were detected using primary antibodies, followed by HRP-conjugated secondary antibodies, and visualized by employing ECL as the HRP substrate. Quantification of immunoblot was performed by ImageJ.

### Colony formation assay

The proliferation potential of cells was assessed by plating 500 cells in 6-well plates and treated with the indicated concentration of flubendazole or vehicle control. After 2 weeks, cells were fixed with methanol and stained with crystal violet. The number of colonies was counted. Data represent the mean ± SD from 3 independent experiments performed in triplicate wells.

### Annexin-V/PI dual staining

The apoptotic ratio was measured by flow cytometry (Becton Dickinson, Franklin Lakes, NJ) after employing an Annexin-V-FLUOS Staining Kit (Roche, Germany). The detailed procedures were performed according to the corresponding manufacturer's instructions.

### Scratch assay

The MDA-MB-231 cells were cultured in 6-well plates and scratch-wounded by sterilized pipettes. Then the cells were washed with PBS and cultured with normal medium or flubendazole. After 24 h incubation, pictures were taken by phase-contrast microscope.

### Transwell migration assay

The MDA-MB-231 cells were resuspended in 24-well culture plates with flubendazole and seeded on transwell filters (8 μm pore size, Millipore). Inoculate serum-free DMEM medium in the top chamber, and add DMEM supplemented with 10% FBS in the bottom chamber. After 12 h, cells on the top side of the filters were wiped by cotton swaps. Cells on the lower side were then fixed in 4% paraformaldehyde and stained with 0.1% crystal violet. Images were taken under an inverted microscope.

### Transfection

si-EVA1A, si-Control, vector, Flag-EVA1A^WT^, Flag-EVA1A^N110A^, Flag-EVA1A^W135A^, Flag-EVA1A^T113A^ were synthesized by Genechem (Shanghai, China). The sequences of the siRNA or cDNA involved in this study listed in Supplementary table 2. si-ATG5 siRNA (6345, CST) and Control siRNA (6568, CST) were purchased from CST. The RNA was transfected with Lipofectamine 3000 reagent (Thermo Fisher Scientific) for 48 h according to the manufacturer's protocol.

### Mice tumor models

Subcutaneous xenograft model: 40 female nude mice (BALB/c, 6-8weeks, 20-22g) were injected with MDA-MB-231 cells (2 × 10^6^) subcutaneously. About a week later, when the tumor size reached 100 mm^3^ in volume (V = L × W^2^/2), the mice were divided into four groups (control group, normal; low dose group, 10mg/Kg/day; middle dose group, 20 mg/Kg/day; high dose group, 40 mg/Kg/day). During the treatment, the weight of mice was recorded daily while the size of the tumor was recorded every three days until the end of the study. The spleen, liver, kidney and tumor tissue were harvested, weighed, and photographed, and then immediately frozen in liquid nitrogen or fixed in formalin for further experiments.

Intravenous xenograft model: 40 female nude mice (BALB/c, 6-8 weeks, 20-22 g) were injected with MDA-MB-231 cells (2 × 10^6^) intravenously. About a week later, the mice were divided into four groups (control group, normal; low dose group, 10 mg/Kg/day; middle dose group, 20 mg/Kg/day; high dose group, 40 mg/Kg/day). During the treatment, the weight of mice was recorded daily until the end of the study. Lung tissue was collected and photographed, and then immediately frozen in liquid nitrogen or fixed in formalin for further experiments.

### RNA-seq data analysis

MDA-MB-231 cells treated with 0.5 μM of flubendazole or control in triplicate for 24 h. RNA purity was checked by the NanoPhotomer® spectrophotometer (IMPLEN, CA, USA). RNA concentration was measured with Qubit® RNA Assay Kit in Qubit®2.0 Flurometer (Life Technologies, CA, USA). RNA integrity was assessed by the RNA Nano 6000 Assay Kit of the Bioanalyzer 2100 system (Agilent Technologies, CA, USA). Sequencing libraries were generated using NEBNext® UltraTM RNA Library Prep Kit for Illumina® (NEB, USA) following manufacturer's recommendations and index codes were added to attribute sequences to each sample. The clustering of the index-coded samples was performed on a cBot Cluster Generation System using TruSeq PE Cluster Kit v3-cBot-HS (Illumia) according to manufacturer's instructions.

Fastq files were aligned to human reference genome hg19 using HiSat with default parameters 49. featureCounts v1.5.0-p3 was used to count the reads numbers mapped to each gene 50. And then FPKM of each gene was calculated based on the length of the gene and reads count mapped to this gene. The analysis of differentially expressed genes (DEGs) between the treatment and control groups was performed using the DESeq2 R package 51. P-values (padj) were adjusted using the Benjamini and Hochberg method 52. A corrected P-value < 0.05 and an absolute log2(fold change) > 1 were set as the threshold for significantly differential expression. GO and KEGG pathway enrichment analyses of the DEGs were performed by using the DAVID 53 and GSEA 54 annotation. For the GO terms and KEGG pathway enrichment analyses, padj < 0.05 was used as a threshold for significant enrichment by DEGs.

### Molecular docking

The protein sequence of EVA1A was used to model its 3D structure by the Robetta server using the Rosetta de novo protocol 55. The binding pocket prediction was carried out from SiteMap Schrodinger suite 56-57. Flubendazole were constructed using the Accelrys Discovery Studio (version 3.5; Accelrys, SanDiego, CA, USA) molecular modeling software and were energy minimized with the CHARMm force field. Docking was performed as previously described 34. The CDOCKER protocol was employed as docking approaches to conduct semi-flexible docking 58.

### Statistical analysis

All the presented data and results were confirmed by at least three independent experiments. The data were expressed as means ± SEM and analyzed with GraphPad Prism 7.0 software. Statistical differences between two groups were determined using Student's t test, while between multiple groups were determined using one-way analysis of variance. *P* < 0.05 was considered statistically significant.

## Supplementary Material

Supplementary figures and table S2.

Supplementary table S1.

## Abbreviations

TNBC: triple-negative breast cancer
RNA-seq: RNA sequencing
IHC: immunohistochemistry
IF: immunofluorescence
IB: immunoblotting
ER: estrogen receptor
PR: progesterone receptor
HER-2: human epidermal growth factor receptor-2
ATG5: autophagy-related 5
3-MA: 3-methyladenine
BafA1: bafilomycin A1
CQ: chloroquine
EVA1A: eva-1 homolog A
TMEM166: transmembrane protein 166
FAM176A: sequence similarity 176 families
