# Supplementary material for: Flubendazole elicits anti-cancer effects via targeting EVA1A-modulated autophagy and apoptosis in Triple-negative Breast Cancer
Source: Theranostics. 2020 Jul 2;10(18):8080–97. doi: 10.7150/thno.43473 (PMC7381743; doi:10.7150/thno.43473)
Supplement: Supplementary file 1 — Supplementary figures and table S2. [file thnov10p8080s1.pdf]

Supplementary Material

Flubendazole elicits anti-cancer effects via targeting EVA1A-modulated autophagy and apoptosis in triple-negative breast cancer

Yongqi Zhen[a, b], Rongyan Zhao[a], Minjuan Wang[a], Xing Jiang[a], Feng Gao[a], Leilei Fu[a*], Lan Zhang [a*], Xian-Li Zhou[a, b*]

[a]School of Life Science and Engineering, Southwest Jiaotong University, Chengdu, P.R. China.

[b]Key Laboratory of Advanced Technologies of Materials, Ministry of Education, Southwest Jiaotong University, Chengdu, P.R. China.

*Correspondence: Dr. Leilei, Fu, E-mail: leilei_fu@163.com; Dr. Lan, Zhang, E-mail: zhanglanx_9@126.com; Prof. Xian-Li, Zhou, E-mail: zhouxl@swjtu.edu.cn.

Postal address: School of Life Science and Engineering, Southwest Jiaotong University, NO.111, North Second Ring Road, Chengdu 610031, Sichuan, P.R. China.

Supplementary Figure S1

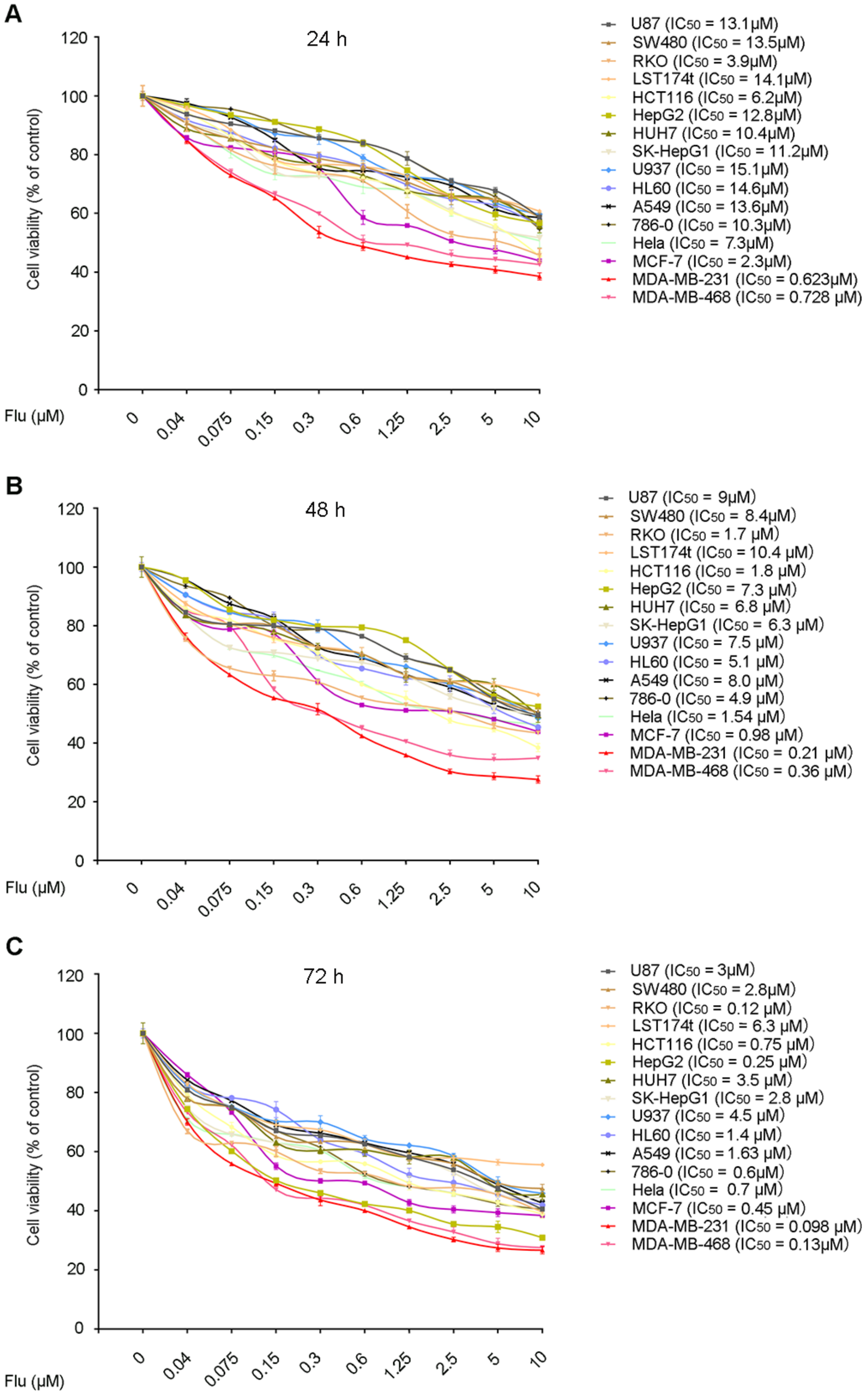

Figure S1. Antitumor effect of flubendazole. (A-C) The cell viability of concentration-dependent and time-dependent treated with flubendazole in various tumor lines. Cell viabilities were

measured by MTT assay, and the IC_{50} values were calculated by Prism 7.0. Data are expressed as mean ± SEM. All data were representative of at least three independent experiments.

Supplementary Figure S2

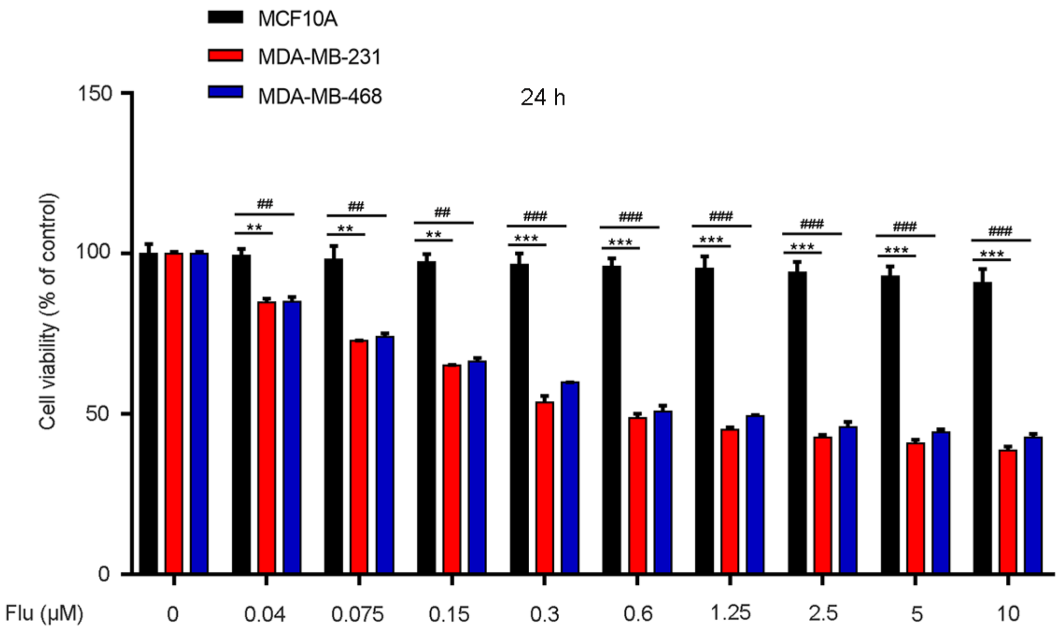

Figure S2. Cell viability of flubendazole on normal breast cells and TNBC cells. (A) The cell viability of concentration-dependent treated with flubendazole for 24 h. Cell viabilities were measured by MTT assay. Data are expressed as mean ± SEM. All data were representative of at least three independent experiments. **, $P < 0.01$, ***, ###, $P < 0.001$. Statistical significance compared with respective control groups.

Supplementary Figure S3

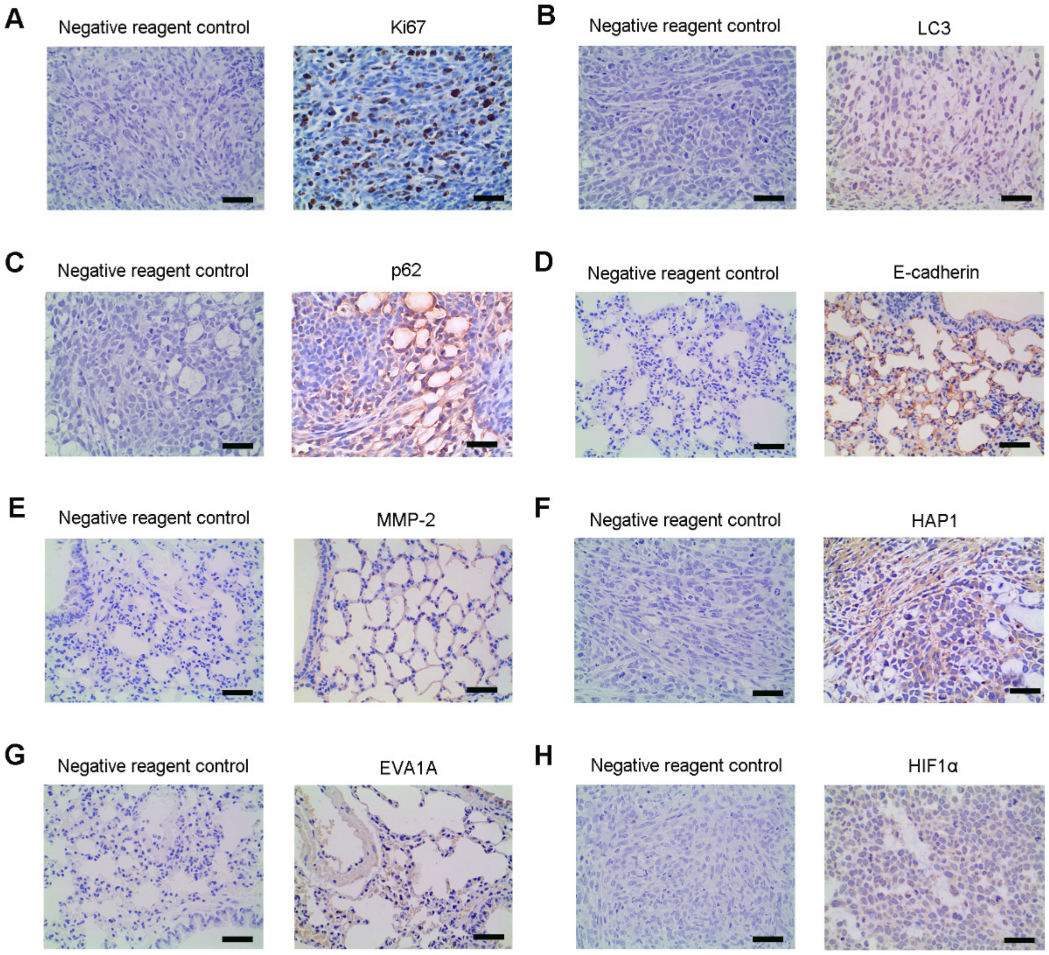

Figure S3. Immunohistochemistry from tumor sections or lung tissue in nude mice. (A-H) Representative images of Ki67, LC3, p62, E-cadherin, MPP-2, EVA1A, HAP1 and HIF1α in nude mice tumor sections or lung tissues and their respective negative controls, which used as the indication of antibody specificity.

Supplementary Figure S4

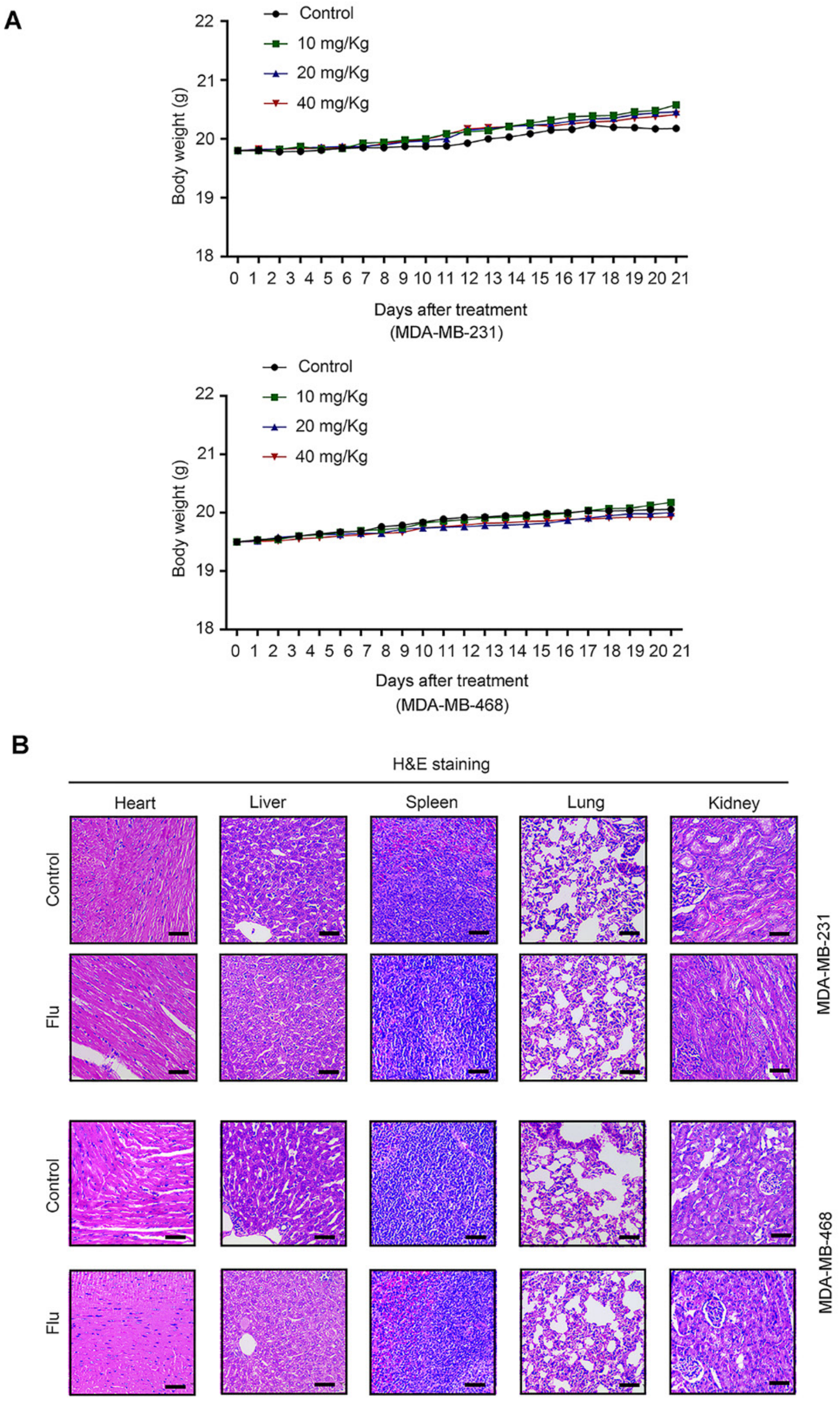

Figure S4. Flubendazole inhibits TNBC *in vivo*. (A) Body weight change of mice in different group, compared with control group. (B) Representative H&E staining in heart, liver, spleen, lung, kidney sections. Scale bar, 40 μm. Data are expressed as mean ± SEM. All data were representative of at least three independent experiments.

Supplementary Figure S5

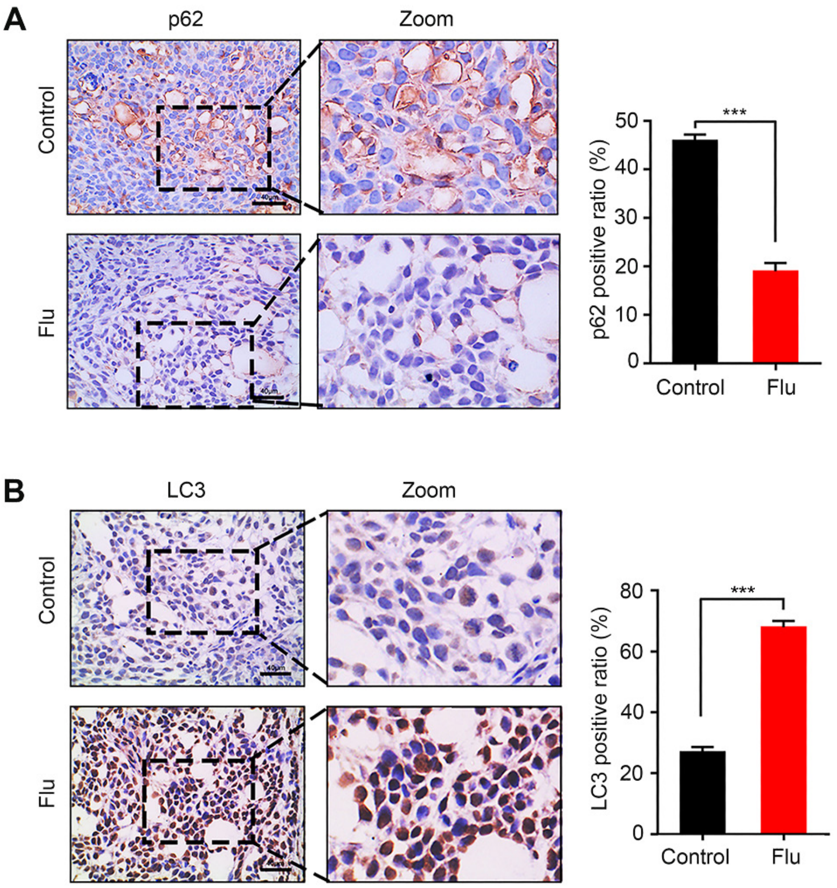

Figure S5. Flubendazole induces autophagy *in vivo*. (A-B) The expression of p62 and LC3 in representative tumor sections of nude mice from control and median dose (20 mg/Kg). Representative images and quantitative analysis of the percentage of positive ratios were shown. Scale bar, 40 μm. Data are expressed as mean ± SEM. All data were representative of at least three independent experiments. *, $P < 0.05$, **, $P < 0.01$, ***, $P < 0.001$. Statistical significance compared with respective control groups.

Supplementary Figure S6

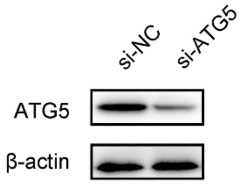

Figure S6. MDA-MB-231 cells were transfected with control or ATG5 siRNA. Then, the expression levels of ATG5 was determined by western blot analysis. β-Actin was measured as the loading control.

Supplementary Figure S7

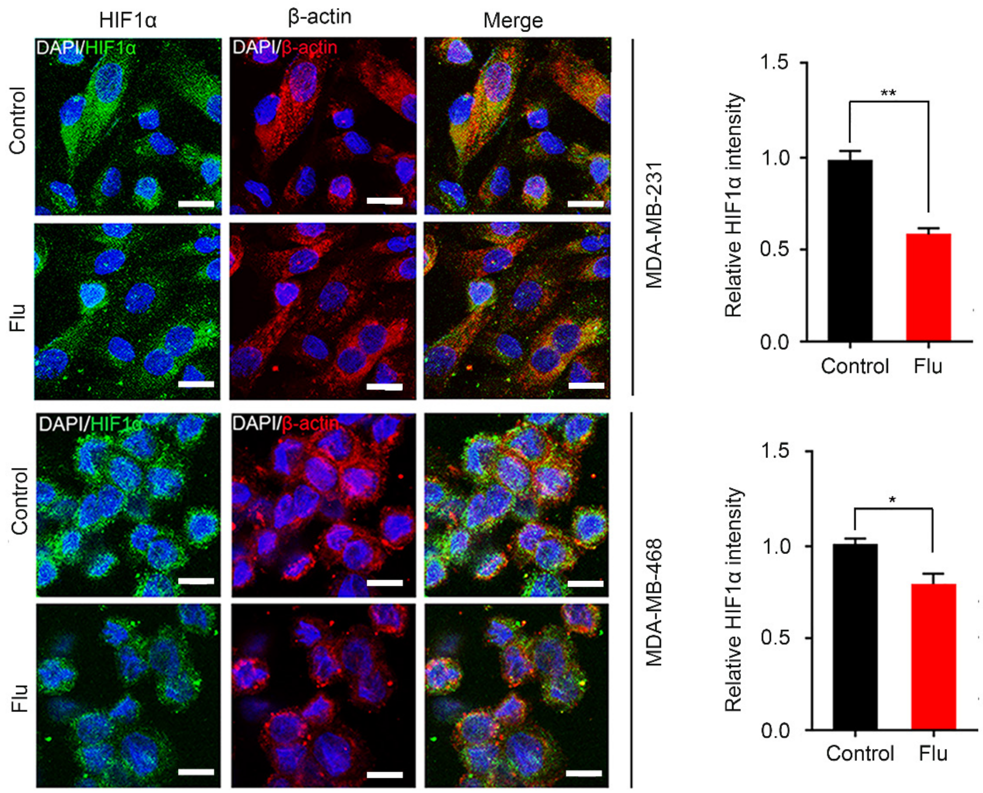

Figure S7. Flubendazole downregulates the expression of HIF1α *in vitro*. Immunofluorescence analysis of HIF1α in MDA-MB-231 and MDA-MB-468 cells treated with or without flubendazole (0.5 μM) for 24 h. Scale bar, 20 μm. Data are expressed as mean ± SEM. All data were representative of at least three independent experiments. *, $P < 0.05$, **, $P < 0.01$, ***, $P < 0.001$. Statistical significance compared with respective control groups.

Supplementary Figure S8

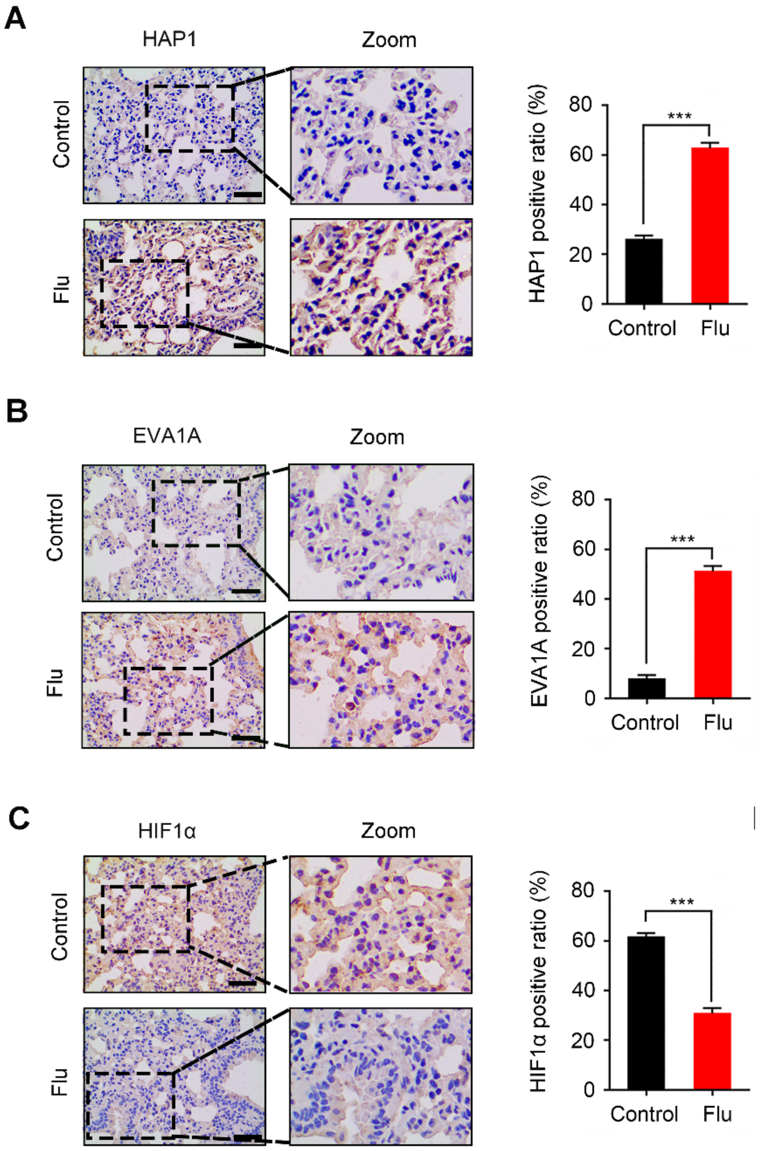

Figure S8. Flubendazole regulates the expression of HAP1, EVA1A and HIF1α *in vivo*. (A-C) The expression of HAP1, EVA1A and HIF1α in representative lung sections of nude mice from vehicle and median dose (20 mg/Kg). Representative images and quantitative analysis of the percentage of positive ratios were shown. Scale bar, 40 μm. Data are expressed as mean ± SEM. All data were representative of at least three independent experiments. *, $P < 0.05$, **, $P < 0.01$, ***, $P < 0.001$. Statistical significance compared with respective control groups.

Supplementary Figure S9

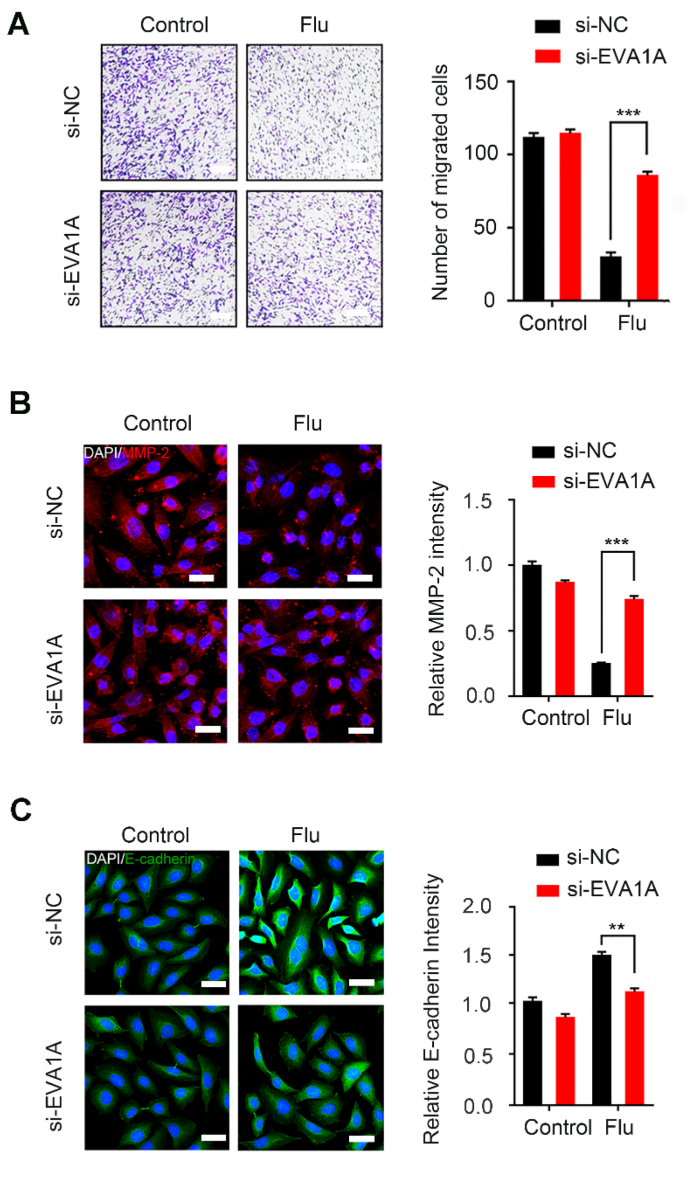

Figure S9. Flubendazole elicits anti-migration potential via targeting EVA1A *in vitro*. (A) The MDA-MB-231 cells were transfected with control or EVA1A siRNA, followed by treatment with or without flubendazole (0.5 μM) for 24 h. Transwell assay was used to measure migration capabilities of cells. Representative images and statistics were shown. Scale bar, 100 μm. (B-C) The MDA-MB-231 cells were transfected with control or EVA1A siRNA, followed by treatment with or without flubendazole (0.5 μM) for 24 h. The expression of MMP-2 and E-cadherin were

analyzed by immunofluorescence. Scale bar, 20 μm. Data are expressed as mean ± SEM. All data were representative of at least three independent experiments. *, $P < 0.05$, **, $P < 0.01$, ***, $P < 0.001$. Statistical significance compared with respective control groups.

Supplementary Table 2. List of siRNA or cDNA sequences

Gene name	siRNA sense sequences (5’-3’)
si-EVA1A	GAGCCTGAATCGCTACTATTA
si-Control	TTCTCCGAACGTGTCACGT

Recombinant DNA	DNA sense sequences
C-terminal Flag-tagged EVA1A^{WT}	ATGAGGCTGCCCCTCAGCCACAGCCCAGAGCACGTGGAGATGGCTTTG<br>CTCAGCAACATCCTAGCGGCCTATTCCTTTGTCTCAGAAAATCCTGAGC<br>GAGCAGCTCTGTACTTTGTTTCTGGCGTGTGCATCGGGCTGGTGCTGAC<br>CCTGGCTGCTCTGGTGATAAGGATCTCTTGCCACACAGACTGCAGGCGG<br>CGTCCCGGGAAGAAGTTCCTGCAGGACAGAGAGAGCAGCAGCGACAG<br>CAGCGACAGCGAGGATGGCAGTGAGGACACCGTGTCCGATCTCTCCGT<br>GCGGAGACACCGCCGCTTCGAGAGGACTTTGAACAAGAATGTGTTCAC<br>CTCTGCGGAGGAGCTGGAGCGCGCCCAGCGGCTGGAGGAGCGCGAGC<br>GCATCATCAGGGAGATCTGGATGAATGGCCAGCCTGAGGTGCCCGGGA<br>CCAGGAGCCTGAATCGCTACTATTAGGACTACAAGGATGACGATGACAA<br>GGATTACAAAGACGACGATAAGGACTATAAGGATGATGACGACAAA
C-terminal Flag-tagged EVA1A^{N110A}	ATGAGGCTGCCCCTCAGCCACAGCCCAGAGCACGTGGAGATGGCTTTG<br>CTCAGCAACATCCTAGCGGCCTATTCCTTTGTCTCAGAAAATCCTGAGC<br>GAGCAGCTCTGTACTTTGTTTCTGGCGTGTGCATCGGGCTGGTGCTGAC<br>CCTGGCTGCTCTGGTGATAAGGATCTCTTGCCACACAGACTGCAGGCGG<br>CGTCCCGGGAAGAAGTTCCTGCAGGACAGAGAGAGCAGCAGCGACAG<br>CAGCGACAGCGAGGATGGCAGTGAGGACACCGTGTCCGATCTCTCCGT<br>GCGGAGACACCGCCGCTTCGAGAGGACTTTGAACAAGGCTGTGTTCAC<br>CTCTGCGGAGGAGCTGGAGCGCGCCCAGCGGCTGGAGGAGCGCGAGC<br>GCATCATCAGGGAGATCTGGATGAATGGCCAGCCTGAGGTGCCCGGGA<br>CCAGGAGCCTGAATCGCTACTATTAGGACTACAAGGATGACGATGACAA<br>GGATTACAAAGACGACGATAAGGACTATAAGGATGATGACGACAAA
C-terminal Flag-tagged EVA1A^{W135A}	ATGAGGCTGCCCCTCAGCCACAGCCCAGAGCACGTGGAGATGGCTTTG<br>CTCAGCAACATCCTAGCGGCCTATTCCTTTGTCTCAGAAAATCCTGAGC<br>GAGCAGCTCTGTACTTTGTTTCTGGCGTGTGCATCGGGCTGGTGCTGAC<br>CCTGGCTGCTCTGGTGATAAGGATCTCTTGCCACACAGACTGCAGGCGG<br>CGTCCCGGGAAGAAGTTCCTGCAGGACAGAGAGAGCAGCAGCGACAG<br>CAGCGACAGCGAGGATGGCAGTGAGGACACCGTGTCCGATCTCTCCGT<br>GCGGAGACACCGCCGCTTCGAGAGGACTTTGAACAAGAATGTGTTCAC<br>CTCTGCGGAGGAGCTGGAGCGCGCCCAGCGGCTGGAGGAGCGCGAGC<br>GCATCATCAGGGAGATCGCGATGAATGGCCAGCCTGAGGTGCCCGGGA<br>CCAGGAGCCTGAATCGCTACTATTAGGACTACAAGGATGACGATGACAA<br>GGATTACAAAGACGACGATAAGGACTATAAGGATGATGACGACAAA
C-terminal Flag-tagged EVA1A^{T113A}	ATGAGGCTGCCCCTCAGCCACAGCCCAGAGCACGTGGAGATGGCTTTG<br>CTCAGCAACATCCTAGCGGCCTATTCCTTTGTCTCAGAAAATCCTGAGC<br>GAGCAGCTCTGTACTTTGTTTCTGGCGTGTGCATCGGGCTGGTGCTGAC<br>CCTGGCTGCTCTGGTGATAAGGATCTCTTGCCACACAGACTGCAGGCGG<br>CGTCCCGGGAAGAAGTTCCTGCAGGACAGAGAGAGCAGCAGCGACAG

	CAGCGACAGCGAGGATGGCAGTGAGGACACCGTGTCCGATCTCTCCGT<br>GCGGAGACACCGCCGCTTCGAGAGGACTTTGAACAAGAATGTGTTCGC<br>CTCTGCGGAGGAGCTGGAGCGCGCCCAGCGGCTGGAGGAGCGCGAGC<br>GCATCATCAGGGAGATCTGGATGAATGGCCAGCCTGAGGTGCCCGGGA<br>CCAGGAGCCTGAATCGCTACTATTAGGACTACAAGGATGACGATGACAA<br>GGATTACAAAGACGACGATAAGGACTATAAGGATGATGACGACAAA
